# *DSR*: enhanced modelling and refinement of disordered structures with *SHELXL*

**DOI:** 10.1107/S1600576715005580

**Published:** 2015-04-25

**Authors:** Daniel Kratzert, Julian J. Holstein, Ingo Krossing

**Affiliations:** a Institute for Inorganic and Analytical Chemistry, Albert-Ludwigs-Universität Freiburg, Albertstrasse 21, Freiburg 79104, Germany; b GZG, Abteilung Kristallographie, Georg-August-Universität Göttingen, Goldschmidtstrasse 1, Göttingen 37077, Germany; c Global Phasing Ltd, Sheraton House, Castle Park, Cambridge CB3 0AX, England

**Keywords:** X-ray structure, refinement, disorder, *SHELXL*, molecular fragments

## Abstract

One of the remaining challenges in single-crystal structure refinement is the proper description of disorder in crystal structures. This paper describes a computer program that performs semi-automatic modelling of disordered moieties in *SHELXL* [Sheldrick (2015 ▶). *Acta Cryst.* C**71**, 3–8.]. The new program contains a database that includes molecular fragments and their corresponding stereochemical restraints, and a placement procedure to place these fragments on the desired position in the unit cell. The program is also suitable for speeding up model building of well ordered crystal structures.

## Abstract 2

The new computer program *DSR* enables semi-automatic modelling of disordered or well ordered moieties in crystal structures *via* a placement procedure of molecular fragments and corresponding stereochemical restraints from a database.

## 1. Introduction   

One of the remaining challenges in applied crystallography is the proper modelling of disorder in crystal structures. We refer here to the term ‘disorder’ as the randomly differing position of some atoms in different unit cells (Müller *et al.*, 2006 ▶; Müller, 2009 ▶). Crystal structures of small molecules and supramolecular systems tend to have disordered solvent molecules or disordered moieties, as illustrated by the summary statistics of the Cambridge Structural Database (CSD; Groom & Allen, 2014 ▶), which include roughly 23% of disordered structures. Although routines and methods to model disorder already exist in *SHELXL* (Sheldrick, 2008 ▶, 2015 ▶), we felt the need to make this procedure more intuitive, faster and more robust, even for large entities and complex cases. Therefore, we developed a new program called *DSR* (disordered structure refinement).

## 2. Technical description and functionality   

**Figure 1:**
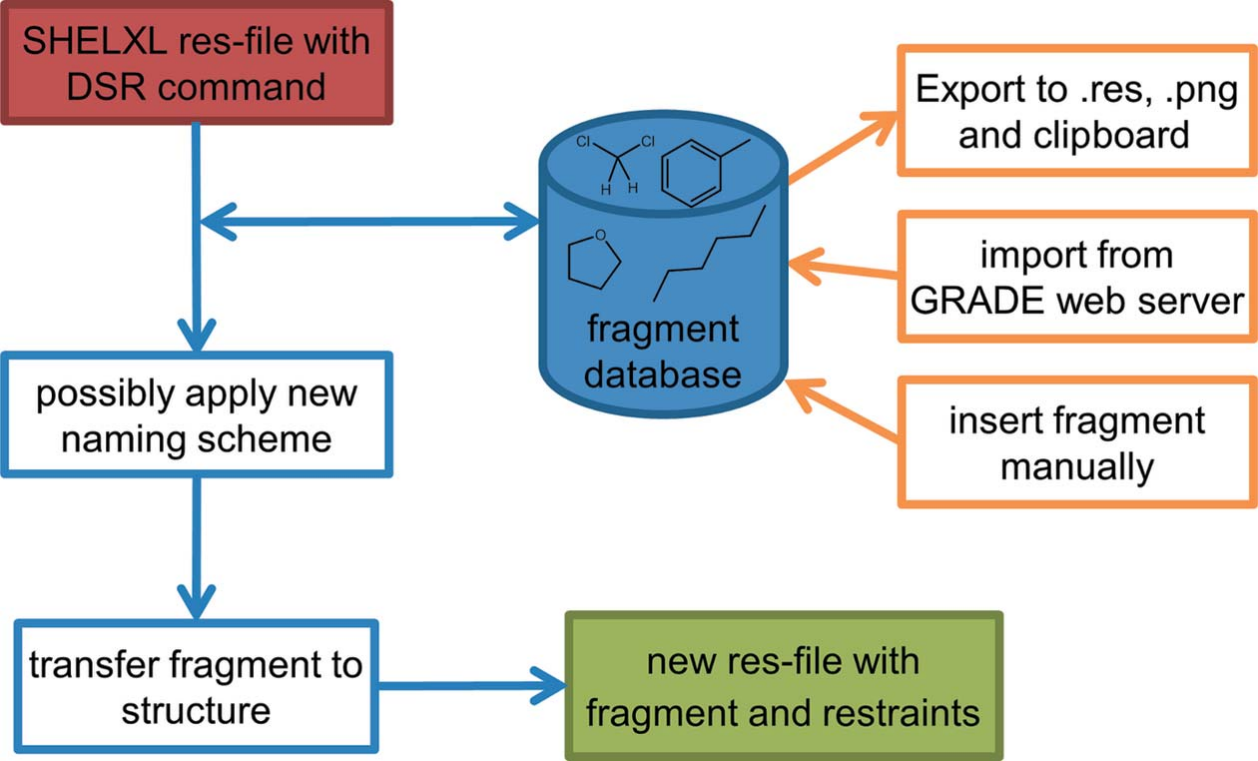
The general work flow of the *DSR* program.

*DSR* is written in Python and is executed *via* the system command line. Its main purpose is the transfer of molecular fragments to a user-defined location in the target structure. Writing a special *DSR* command into the *SHELXL* .res file of the target structure instructs *DSR* on where to place and how to orient a molecular fragment from the fragment database in the unit cell (Fig. 1 ▶). In addition, it is possible to define the occupancy, residue or part number of the inserted fragment. The fragment can be inverted, *e.g.* to fit the second conformer of twisted tetrahydrofuran. If desired, automatically calculated distance restraints for all bonds in the molecular fragment can replace the restraints obtained from the database. Additional options in the system command line allow the import and export of molecular fragments to and from the database, and listing of and searching for database content.

### 2.1. Database   

Since there are a number of molecules and functional groups that are regularly involved in disorder, we compiled a database for *DSR* with common molecular fragments similar to Guzei’s ‘Idealized Molecular Geometry Library’ (Guzei, 2014 ▶). Database entries contain the ideal starting geometry of a fragment, together with a corresponding stereochemical restraint dictionary (Evans, 2007 ▶; Konnert & Hendrickson, 1980 ▶), which typically consists of restraints for 1,2-distances (bonds) and 1,3-distances (equivalent to bond angles) for use in *SHELXL*. They are defined either by means of chemical equivalence (SADI) or as specific target values (DFIX, DANG). If applicable, information on planar groups of atoms is also included (FLAT). For now, the standard uncertainties (s.u.s) of the restraints come from the database and they are simply SIMU/RIGU [first atom] > [last atom]. In future versions, it might be possible to calculate suitable s.u.s during fragment transfer or to analyse the results of the *SHELXL* refinement to adapt the s.u.s to the respective structural environment.

The database is in ASCII text format and has been intentionally kept simple to be human-readable and -writable, which allows manual editing and extension of the database. The database entries provided with *DSR* include no hydrogen atoms (except for water), as the authors consider *SHELXL* constraints (AFIX) to be more robust and reliable for H-atom treatment in disordered moieties. Since every restraint command valid for *SHELXL* (including HFIX) is supported in the *DSR* database, appropriate riding H atoms can easily be included in a database entry by placing its respective HFIX command in the respective database entry (see Table 1 ▶). Such modifications should not be made in the main database provided with *DSR* (dsr_db.txt) but rather in the user database (dsr_usr_db.txt). This will never be overwritten during a program update and should therefore be used by users to store additional entries.

The database contains over 70 entries of commonly dis­ordered solvent molecules and molecular fragments, such as tetrahydrofuran, toluene, dichloromethane, *tert*-butyl groups, hexane and the tosylate anion, and also less common mol­ecules like supramolecular ligands (Pascu *et al.*, 2014 ▶; Ronson *et al.*, 2013 ▶; Ramadhar *et al.*, 2015 ▶) and molecules that have been soaked into porous metal–organic framework complexes (Inokuma *et al.*, 2013 ▶). The complete list at the time of writing is available in the supporting information (the pictures therein were drawn using *Jmol*; http://www.jmol.org/).

### 2.2. Import/export   

New fragments from quantum mechanical calculations, experimentally determined crystal structures or structural databases like the CSD can be incorporated into the *DSR* database by manual addition of a new database entry. *DSR* accepts both Cartesian and fractional atomic coordinates. Another possibility for input is the fully automated import of fragments from the *GRADE Web Server* of Global Phasing Ltd (Smart & Womack, 2014 ▶). *GRADE* is a ligand restraint generator included in *BUSTER* (Bricogne *et al.*, 2011 ▶), and its main source of restraint information is the CSD queried using the *MOGUL* program (Bruno *et al.*, 2004 ▶) developed by the Cambridge Crystallographic Data Centre. Whenever small-molecule information is not available, *GRADE* uses quantum chemical procedures to obtain the restraint values.

Fragments from the database can be exported to a *SHELXL* .res file and a portable net graphics (.png) file to inspect the atoms of a fragment and their labelling scheme [utilizing *PLATON* (Spek, 2009 ▶) and *ImageMagick* (http://www.imagemagick.org) if installed]. A fragment can also be copied to the computer clipboard to be used in the *Olex2* ‘fit mode’ (Dolomanov *et al.*, 2009 ▶; Guzei, 2014 ▶).

### 2.3. Transfer and refinement   

**Figure 2:**
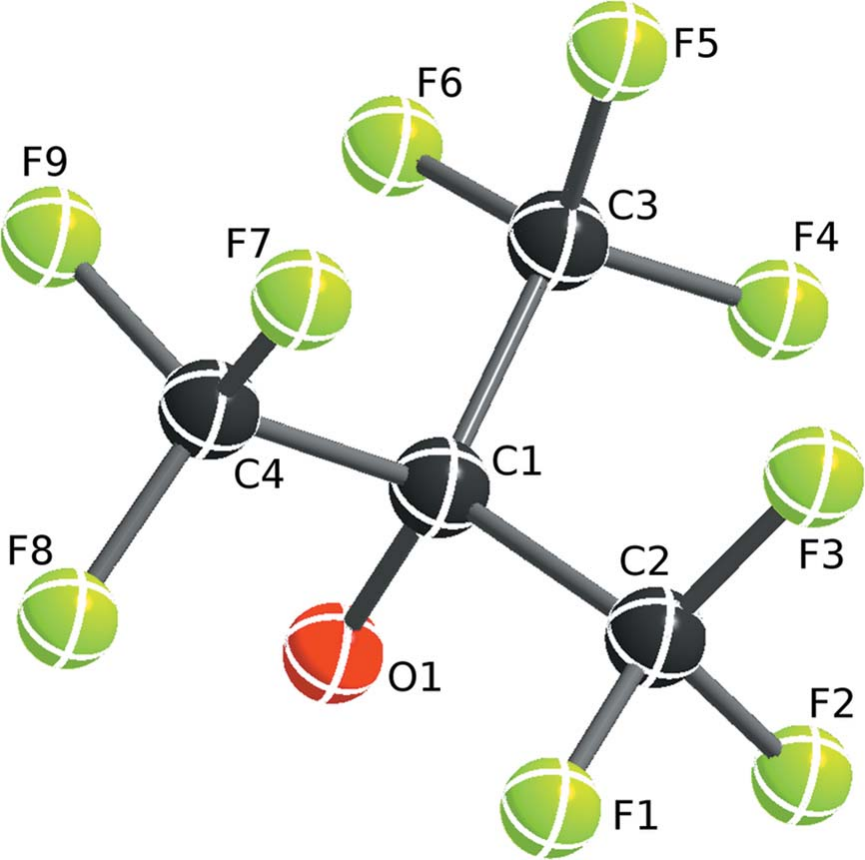
The numbering of the central atoms of the OC(CF_3_)_3_ fragment from the *DSR* fragment database. The molecular diagrams here and in subsequent figures were created using *ShelXle* (Hübschle *et al.*, 2011 ▶).

*DSR* leaves the definition of disorder to the user and automates the time-consuming transfer and tedious restraining part. The main concept of *DSR* is similar to what is nowadays commonly applied in macromolecular crystallography, where a complete molecular fragment (see *e.g.* Figs. 2 or 5 below) is modelled instead of single atoms. The fitted fragment should contain at least three atoms.

Before going into more detail about how *DSR* works, we summarize briefly the manual steps typically performed when modelling a second conformer in *SHELXL* without *DSR*.

(1) Find and label all *Q* peaks corresponding to the secondary position of atoms in the difference Fourier map. Sometimes this requires several intermediate refinement steps.

(2) Assign a free variable to the site occupancy for both disordered components.

(3) Define restraints for bond lengths, bond angles and possibly anisotropic displacement parameters.

Using *DSR*, the above steps require one command line to be written into the *SHELXL* .res file. In practice, the user defines a minimum of three non-collinear target positions (occupied by atoms or *Q* peaks in the structure) and the corresponding atoms from the database fragment (source atoms) intended to be placed on the target positions. *DSR* then automatically generates the required input for the fitting procedure in *SHELXL* (FRAG/FEND) that is needed to place the requested fragment on the desired position in the unit cell. Afterwards, the *SHELXL* input file is prepared for a full refinement employing automatically adapted restraints from the *DSR* database.

The stereochemical restraints applied by *DSR* are capable of stabilizing poor starting models and thereby enhancing the least-squares minimization to move towards the global minimum. They are also capable of stabilizing moieties with low occupancy factors. Nevertheless, it is advisable for the user to check whether the restraints are physically sensible (Biadene, 2006 ▶; Müller, 2009 ▶). The applied restraints can also be manually softened or removed in a subsequent refinement step, if necessary, after considering the refinement stability. Detailed descriptions of practical examples of disorder modelling and refinement strategies in *SHELXL* can be found in the excellent *Crystallographer’s Guide to *SHELXL** (Müller *et al.*, 2006 ▶).

Although *DSR* has been primarily defined as an aid in disorder modelling, it can also be employed for rigid-body refinements and to enhance model building of large and complex supramolecular structures sharing typical characteristics with macromolecular structures, *i.e.* a high degree of structural flexibility paired with a limited resolution of the experimental data. *DSR* has already been successfully employed in that context (Schouwey *et al.*, 2014 ▶; Wise *et al.*, 2015 ▶; Wood *et al.*, 2015 ▶).

### 2.4. Two worked examples   

Modelling of a perfluorinated *tert*-butyl group (Fig. 2 ▶) serves as a typical example where *DSR* greatly facilitates structure refinement of a 14-atom entity. The disorder of this group is frequently observed in the [Al(O*R* ^F^)_4_]^−^ type of weakly coordinating counter-ion (Krossing, 2001 ▶; Bihlmeier *et al.*, 2004 ▶; Köchner *et al.*, 2012 ▶).

**Figure 3:**
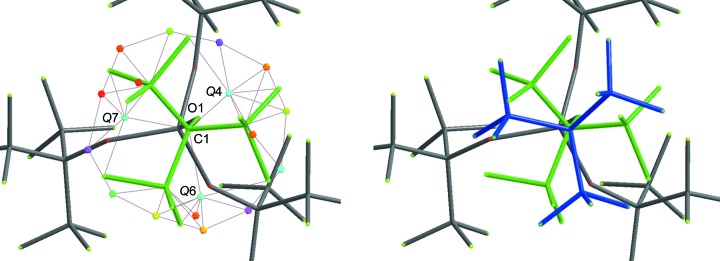
(Left) A stick representation of [Al(O*R* ^F^)_4_]^−^. The disordered perfluorinated *tert*-butyl OC(CF_3_)_3_ moiety is highlighted in green. Its second position is indicated by maxima in the difference Fourier density map (*Q* peaks), which are shown as coloured icosahedra. (Right) The second position of the disordered OC(CF_3_)_3_ moiety (blue), successfully placed using *DSR*.

The second position of the OC(CF_3_)_3_ moiety in Fig. 3 ▶ (left) (Lichtenthaler *et al.*, 2013 ▶) is located near the positions of atoms O1 and C1 and the maxima in the difference Fourier density map (*Q* peak positions) of *Q*4, *Q*7 and *Q*6. These three *Q* peaks correspond to atoms C2, C3 and C4 in the database fragment (Fig. 2 ▶). Fortunately, the F atoms do not have to be placed explicitly, as the subsequent refinement with restraints allows the rotation of the CF_3_ groups to the correct geometry.

*DSR* currently provides two options for placing fragments. The command PUT places the fragment on a desired position, leaving all target atoms unaffected. Alternatively, REPLACE replaces all target atoms. After PUT or REPLACE, the user gives the fragment name. To model and refine the second position of the OC(CF_3_)_3_ fragment requires the following *DSR* command line anywhere in the *SHELXL* .res file:

REM DSR PUT OC(CF3)3 WITH O1 C1 C2 C3 ON O1_1 C1_1 Q4 Q7 Q6 PART 2 OCC -21 RESI

Note that the REM at the beginning of the line prevents *SHELXL* from accidently interpreting the *DSR* command. The .res file can now be processed by *DSR* to insert the fragment by executing the following on the command line:

dsr -r filename.res

After successful fragment placement with *DSR*, the resulting structure includes all atoms of the second position of the disordered OC(CF_3_)_3_ moiety (Fig. 3 ▶, right). Atom names, site-occupancy factors and the free variable are automatically assigned. The second disordered part (PART 2), the instructions for residue class and number (RESI 4 CCF3), and the restraints for bond distances and displacement parameters to stabilize the subsequent refinement are also established. Furthermore, *DSR* introduces similarity restraints for the OC(CF_3_)_3_ moiety. Note the fairly large standard deviation of the 1,3-distance restraints for the CF_3_ groups which allow a small tilt:

SADI_CCF3 0.02 C1 C2 C1 C3 C1 C4

SADI_CCF3 0.02 F1 C2 F2 C2 F3 C2 F4 C3 F5 C3 F6 C3 F7 C4 F8 C4 F9 C4

SADI_CCF3 0.04 C2 C3 C3 C4 C2 C4

SADI_CCF3 0.04 O1 C2 O1 C3 O1 C4

SADI_CCF3 0.04 F1 F2 F2 F3 F3 F1 F4 F5 F5 F6 F6 F4 F7 F8 F8 F9 F9 F7

SADI_CCF3 0.1 F1 C1 F2 C1 F3 C1 F4 C1 F5 C1 F6 C1 F7 C1 F8 C1 F9 C1

**Figure 4:**
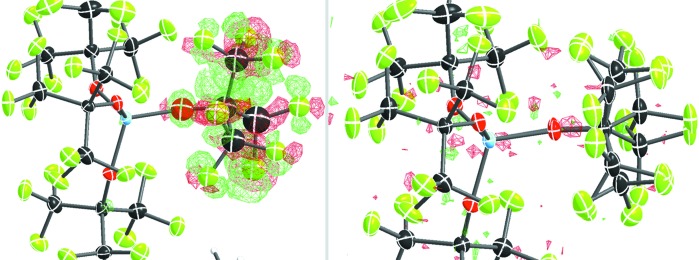
The residual density maps around the disordered OC(CF_3_)_3_ group (left) before and (right) after treatment with *DSR*. The residual electron density is shown as a green (positive) or red (negative) mesh, with isolevels at ±0.70 e Å^−3^ (left) and ±0.30 e Å^−3^ (right).

All other changes made to the .res file can be found in the supporting information. Fig. 4 ▶ illustrates a successful fragment placement and a robust disorder model with an occupancy of 0.44, which significantly improves the residual density.

**Figure 5:**
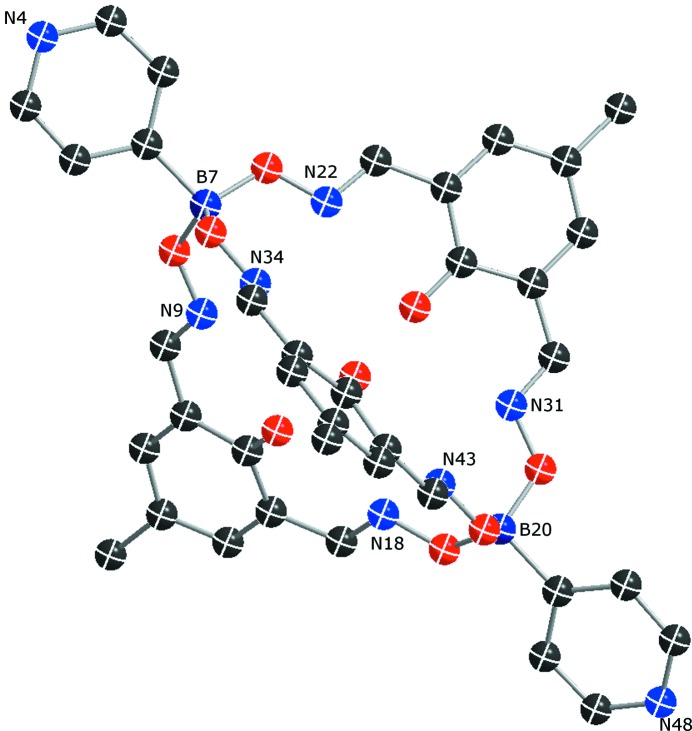
The numbering of the B and N atoms of the clathrochelate ligand fragment (numbered pm41) from the *DSR* fragment database.

Modelling of a disordered three-dimensional supramol­ecular coordination network is a second example to demonstrate the advantages of using *DSR* in a complex structure refinement. The structure consists of supramolecular clathrochelate ligands with a zinc ion in the centre, connected *via* cadmium ions to form a network (Pascu *et al.*, 2014 ▶). In this case, 64% of the main-residue disorder is triggered by the disorder of the Cd ions over a special position (inversion centre) to which the clathrochelate ligands are coordinated. The stereochemical restraints for the organic part of the clathrochelate ligands were generated using *GRADE* and imported into the *DSR* database (Fig. 5 ▶).

**Figure 6:**
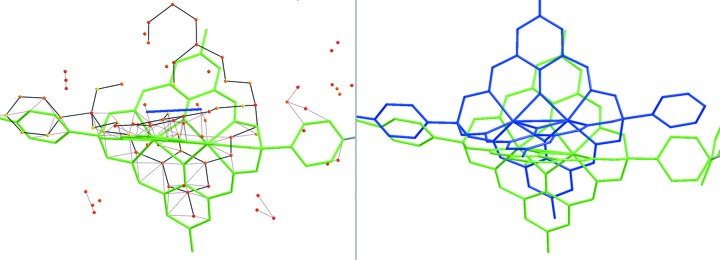
(Left) A stick representation of the coordination network of the example metal–organic framework compound. The clathrochelate moiety is highlighted in green. Its second position is indicated by maxima in the difference Fourier density map (*Q* peaks), which are shown as coloured icosahedra. (Right) The second position of the disordered clathrochelate moiety (blue), successfully placed using *DSR*.

After setting the site-occupancy factor of the first modelled part (PART 1) of the clathrochelate to 0.50, the remaining *Q* peaks clearly show the second clathrochelate position (Fig. 6 ▶, left). The central Zn^2+^ cations of the clathrochelate PART 2 were added manually.

The *DSR* placement of the second part of the clathrochelate entity works best if four (or more) reference points (*Q* peaks) are defined, to ensure a good starting position and orientation for the initial refinement. With the following *DSR* command, all 56 non-H atoms of the second clathrochelate part are introduced at the same time for subsequent refinement by *SHELXL*:

REM DSR PUT PM41 WITH N4 B7 N31 B20 ON Q7 Q133 Q44 Q171 PART 2 OCC 10.5 = RESI PM4

After this modelling step, *R*_1_ dropped by 0.05 to about 0.25, which is also reflected in the decrease in residual density in the modelled region.

## 3. Concluding remarks   

*DSR* is a powerful program to model disorder of medium-sized molecular fragments. It only requires a basic crystallographic background and spares its users most of the tedious work. It is flexible enough to be used with all existing refinement graphical user interfaces (GUIs). Although its main application will probably remain the modelling of disorder, *DSR* will also be beneficial for cases of chemical crystallography belonging to the macromolecular domain, for which a large number of repeating structural motifs need to be modelled and refined against data of limited resolution. Its application has been very promising, especially in the context of supramolecular chemistry. The fitting of ligands to previously identified binding sites of biological macromolecules and the modelling of structures measured at high pressure are possible fields for future applications.

## 4. Program availability   

*DSR* runs on every platform that can run Python (versions 2 and 3), such as Windows, Linux and Mac OS X. It can run as a standalone command line program or can be integrated as a Python module within a GUI. The software is distributed under a free BSD-like license (Kratzert, 2015 ▶). The Windows installation file is created with the Inno Script Studio installer compiler (Kymoto, 2014 ▶). The Linux packages are regular .rpm and .deb files for Redhat- or Debian-based Linux distributions, respectively. The *NetworkX* software library (Hagberg *et al.*, 2013 ▶) is supplied with the Windows installer and Linux program packages. *DSR* can be used together with any GUI like *ShelXle* (Hübschle *et al.*, 2011 ▶) or *WinGX* (Farrugia, 2012 ▶).

The program executables and source files and a detailed user manual can be downloaded from the program’s web page at https://www.xs3.uni-freiburg.de/research/dsr. The development platform is located at https://github.com/dkratzert/DSR.

## Supplementary Material

Supporting information file. DOI: 10.1107/S1600576715005580/fs5104sup1.pdf

## Figures and Tables

**Table 1:** Syntax of the *DSR* database dsr_db.txt and dsr_usr_db.txt The complete syntax is explained in the supporting information.

name>	Start tag
RESI class	Required; defines the residue name of the database entry
restraints	Any restraints and comments, following the *SHELXL* syntax
FRAG 17 a b c	FRAG card with AFIX number and cell parameters
Atom number x y z	One isotropic atom per line, following the *SHELXL* syntax, except that a negative SFAC number means the atom type as atomic number
/name>	End tag; the same as the start tag but with a slash
