# Supplementary material for: DSR: enhanced modelling and refinement of disordered structures with SHELXL
Source: J Appl Crystallogr. 2015 Apr 25;48(Pt 3):933–8. doi: 10.1107/S1600576715005580 (PMC4453980; doi:10.1107/S1600576715005580)
Supplement: Supplementary file 1 [file j-48-00933-sup1.pdf]

Supplementary material for
DSR – Enhanced modelling and refinement of disordered structures with SHELXL

Daniel Kratzert[1*], Julian J. Holstein[2,3], Ingo Krossing[1].

[1] Institute for Inorganic and Analytical Chemistry, Albert-Ludwigs-Universität Freiburg, Albertstraße 21, 79104 Freiburg, Germany, dkratzert@gmx.de.

[2] GZG, Abteilung Kristallographie, Georg-August-Universität Göttingen, Goldschmidtstr. 1, 37077 Göttingen.

[3] Global Phasing Ltd., Sheraton House, Castle Park, Cambridge CB3 0AX, England

A more detailed and regularly updated program manual can be found on the programs main web site https://www.xs3.uni-freiburg.de/research/dsr or the development platform https://github.com/dkratzert/DSR.

Program Installation

The Windows installer (Kymoto, 2014) creates the desired install directory and copies the program files in this directory. A start menu entry is generated with a link to both the user and main database as well as a link to the user manual. Additionally, the program directory is written to the systems PATH and DSR_DB_DIR variable. The Windows installer includes a stripped-down version of Python and the NetworkX library.
The Linux program package (.rpm or .deb) creates the directory /opt/DSR and copies the program files in this directory. Additionally, a file called dsr.sh is copied into the system folder /etc/profile.d which sets the PATH variable and DSR_DB_DIR. The Linux version expects Python version ≥2.7.0 to be installed. The NetworkX library is supplied with the program package.

Required SHELXL version

A recent version of SHELXL is required to work with DSR. Supported are all versions above SHELXL-2012. Recent versions of SHELXL can be downloaded free of charge for academic users from the authors web site: http://shelx.uni-ac.gwdg.de/SHELX DSR searches the system PATH for executables with names shelxl(.exe) or xl(.exe) and uses the one which is found first.

Program Commands

The DSR command has the following syntax:

```
REM DSR PUT/REPLACE fragment WITH atom1 atom2 atom3 ... ON atom2 atom3 atom4 ... PART n OCC
mn RESI class num [alias] DFIX
```

The command is introduced with a REM because SHELXL should never interpret the DSR command line.

`PUT`	Put the fragment on there, ignoring atoms on this position.
`REPLACE`	Replace the target atoms. Hydrogen atoms bond to a replaced target atom are also removed.
`fragment`	The name of the desired molecule or fragment.
`WITH`	Behind WITH are the source atoms, which must be at least three atoms from the fragment.
`ON`	Behind ON are the target atoms, which can either be atoms or q-peaks in the .res- file.

`[atom n]`	Minimum three atoms each (including Q-peaks). Source and target atoms have to include the same number of atoms or Q-peaks. Target atoms can be either regular atoms or atoms in residues. Atoms in residues can be addressed by the "_" notation. C1_2 would be atom C1 in residue number 2.
`PART n`	Optional SHELXL PART definition.
`OCC mn`	Optional occupancy and free variable definition for the fragment.
`DFIX`	Optional, generates DFIX/DANG restraints instead of those from the database. All 1,2- and 1,3-distances in the fragment and their neighboring atoms are restrained with DFIX and DANG respectively. Bonds to symmetry equivalent atoms are ignored.
`RESI class num [alias]`	Optional residue definition as in SHELXL.

Use of Residues

To use the RESI command in DSR has several advantages. It places the fragment into a residue and therefore no renaming of the atoms in the fragment is performed by DSR. If residues are used, the restraints like "SADI_class Atoms" are inserted only once, since they act on the atoms in all residues with the same class. Residues are especially useful, if the same moiety is repeated several times in a crystal structure. Different moieties of the same residue class are distinguished by different residue numbers. Residue number must be unique in a .res file. The DSR command RESI without any further options is normally the best practice. DSR then uses the residue class name from the database and assigns the lowest unused residue number automatically. But the user can also specify a particular residue class and/or number after the RESI command, if desired.

The `RESI` option can be used in three ways:

1) If only a `RESI` command is given (best practice), the residue class is taken from the database entry and the residue number is automatically generated.
2) If `RESI` with only a number is given, DSR takes the residue class from the database with the given number.
3) `RESI` with a number and a class overwrites the information from the database and gives complete control over the residue.

Residues in structure refinement

The concept of residues is well established in structure refinements of macromolecules. Although it simplifies bookkeeping and restraining of complex structures, it is rarely employed by chemical crystallographers, who are mostly dealing with small molecule structures. For using the `RESI` command of DSR, the user should have at least basic knowledge about the concept of residues in SHELXL. The manual on the SHELX website provides more detailed information: http://shelx.uni-ac.gwdg.de/SHELX/wikis.php

Employing identical atomic labelling schemes for several different parts and residues of a structure in a SHELXL refinement only requires a single restraint dictionary to restrain all occasions of that residue class in the same way, which significantly supports handling of large and complex structures in a consistent manner. As small molecule programs written ages ago were initially not designed for the residue concept it should be emphasized that the most elegant way for producing a crystallographic information file (CIF) suitable for other programs (e.g. CHECKCIF) from such a refinement is the "ACTA TABS" command (applies only to SHELXL versions 2014 or later). Additionally, disordered residues should be numbered in between 1 to 9. Atoms names in the resulting CIF can otherwise contain more characters than expected by some programs. For more details please see the SHELXL instructions at http://shelx.uni-ac.gwdg.de/SHELX/shelxl_html.php#ACTA.

Further Details to the $OC(CF_3)_3$ Fragment Placement

Disorder of a perfluorinated *tert*-butyl group is frequently observed in the $[Al(OR^F)_4]^-$ type of weakly coordinating counterions (Krossing, 2001; Bihlmeier *et al.*, 2004; Köchner *et al.*, 2012). CCDC 936462 was first published in (Lichtenthaler *et al.*, 2013) and contains the full crystallographic data of the structure chosen to demonstrate placement of the second $OC(CF_3)_3$ fragment on the second position using DSR. The following command line would be needed in the SHELX .res file:

```
REM DSR PUT OC(CF3)3 WITH O1 C1 C2 C3 ON O1_1 C1_1 Q4 Q7 Q6 PART 2 OCC -21 RESI
```

A disordered fragment requires a free variable for the site occupancy and a part number, which is accomplished by the `OCC` and `PART` commands. For example `OCC 21` sets the free variable number 2 for the fragment and the occupancy to one. Non-existing free variables are introduced to the FVAR instruction automatically.

DSR inserts the following two entries into the res file. The FRAG ... FEND block defines the transferred fragment through the cell parameters and atom coordinates. The other text block beginning with RESI defines the residue, the part, the free variable for the site occupancy and the position of the fitted fragment. The complete fitting process takes less than a second, even on slow computers.

```
FRAG  17  1  1  1  90  90  90
O1    3     -0.014530      1.665900      0.109660
C1    1     -0.001460      0.268140      0.063510
C2    1     -1.133410     -0.232470     -0.907300
F1    4     -2.346610     -0.112730     -0.345440
F2    4     -0.962540     -1.506650     -1.290800
F3    4     -1.122630      0.550280     -2.017630
C3    1      1.405660     -0.231790     -0.431310
F4    4      2.385290      0.423400      0.205610
F5    4      1.532560      0.038430     -1.755380
F6    4      1.578330     -1.551530     -0.250350
C4    1     -0.278130     -0.216050      1.527950
F7    4      0.806020     -0.037590      2.304310
F8    4     -0.589100     -1.528590      1.534600
F9    4     -1.293230      0.469630      2.067350
FEND

[...]

RESI 4 CF3
SADI_CF3 0.02 C1 C2 C1 C3 C1 C4
SADI_CF3 0.02 F1 C2 F2 C2 F3 C2 F4 C3 F5 C3 F6 C3 F7 C4 F8 C4 F9 C4
SADI_CF3 0.04 C2 C3 C3 C4 C2 C4
SADI_CF3 0.04 O1 C2 O1 C3 O1 C4
SADI_CF3 0.04 F1 F2 F2 F3 F3 F1 F4 F5 F5 F6 F6 F4 F7 F8 F8 F9 F9 F7
SADI_CF3 0.1 F1 C1 F2 C1 F3 C1 F4 C1 F5 C1 F6 C1 F7 C1 F8 C1 F9 C1
PART 2 -31
AFIX 179
O1   3     0.081800     0.235411     0.399962   11.000   0.04
C1   1     0.033372     0.232537     0.337248   11.000   0.04
C2   1     0.024600     0.306100     0.306300   11.000   0.04
F1   4     0.000000     0.000000     0.000000   11.000   0.04
F2   4     0.000000     0.000000     0.000000   11.000   0.04
F3   4     0.000000     0.000000     0.000000   11.000   0.04
C3   1     0.122500     0.190800     0.297100   11.000   0.04
F4   4     0.000000     0.000000     0.000000   11.000   0.04
F5   4     0.000000     0.000000     0.000000   11.000   0.04
F6   4     0.000000     0.000000     0.000000   11.000   0.04
C4   1    -0.109100     0.204000     0.333000   11.000   0.04
F7   4     0.000000     0.000000     0.000000   11.000   0.04
F8   4     0.000000     0.000000     0.000000   11.000   0.04
F9   4     0.000000     0.000000     0.000000   11.000   0.04
AFIX 0
PART 0
RESI 0
```

For smaller molecules in lower occupancy or diffuse environment, the use of DFIX bond restraints might be useful. Therefore, the DSR command `DFIX` would ignore all restraints in the database and generate DFIX/DANG/FLAT restraints for all 1,2- and 1,3-bond distances occurring in the fragment and its neighboring atoms.

Further Details on the Supramolecular Clathrochelate Fragment (PM41) Placement

In this structure 64% of main residue disorder is triggered by the disorder of Cd ions over a special position (inversion centre) to which the supramolecular Clathrochelate ligands are coordinated. CCDC 980636 contains the full crystallographic data of this structure, which was first published as MOF **14** in (Pascu *et al.*, 2014). To place the second PM41 fragment on the second position with DSR, the following DSR command line would be needed in the SHELX .res file:

```
REM DSR PUT PM41 WITH N4 B7 N31 B20 ON Q7 Q133 Q44 Q171 PART 2 OCC 10.5 =
   RESI PM4
```

In this case of disordered near a special position the occupancy of each of the two parts must be 0.5 (50%), which is accomplished by the OCC 10.5 command. After fragment placement (FRAG ... FEND) and lifting of constraints from the initial body refinement (AFIX), DSR also writes the following list of stereochemical restraints from the DSR database. As the list contains 206 lines for such a large fragment, all restraints can be written into a text file in the same folder named "dsr_PM41.dfix", which is automatically included in the SHELX .res file by DSR via the "+dsr_PM41.dfix" instruction. "_PM41" is a reference to all residues named "PM4", independent of residue number or PART number. Using the identical atomic labelling scheme in all residues and parts of all PM4 moieties ensures that only one restraint dictionary is required for all of them. (see above +section "Residues in structure refinement")

Restraints in the "dsr_PM41.dfix" file:

```
DFIX_PM4 1.398 0.009 C1 C2
DFIX_PM4 1.598 0.009 B7 C1
DFIX_PM4 1.398 0.009 C1 C6
DFIX_PM4 1.381 0.012 C2 C3
DFIX_PM4 1.333 0.011 C3 N4
DFIX_PM4 1.333 0.011 C5 N4
DFIX_PM4 1.381 0.012 C5 C6
DFIX_PM4 1.408 0.030 B7 O33
DFIX_PM4 1.416 0.030 B7 O8
DFIX_PM4 1.420 0.030 B7 O21
DFIX_PM4 1.325 0.030 N9 O8
DFIX_PM4 1.272 0.007 C10 N9
DFIX_PM4 1.455 0.012 C10 C11
DFIX_PM4 1.431 0.013 C11 C12
DFIX_PM4 1.397 0.011 C11 C16
DFIX_PM4 1.431 0.013 C12 C13
DFIX_PM4 1.267 0.029 C12 O12A
DFIX_PM4 1.455 0.012 C13 C17
DFIX_PM4 1.397 0.011 C13 C14
DFIX_PM4 1.386 0.010 C14 C15
DFIX_PM4 1.509 0.011 C15 C15A
DFIX_PM4 1.386 0.010 C15 C16
DFIX_PM4 1.272 0.007 C17 N18
DFIX_PM4 1.322 0.030 N18 O19
DFIX_PM4 1.424 0.030 B20 O19
DFIX_PM4 1.415 0.030 B20 O44
DFIX_PM4 1.598 0.009 B20 C45
DFIX_PM4 1.417 0.030 B20 O32
DFIX_PM4 1.318 0.030 N22 O21
DFIX_PM4 1.272 0.007 C23 N22
DFIX_PM4 1.455 0.012 C23 C24
DFIX_PM4 1.431 0.013 C24 C25
DFIX_PM4 1.397 0.011 C24 C29
DFIX_PM4 1.267 0.029 C25 O25A
DFIX_PM4 1.431 0.013 C25 C26
DFIX_PM4 1.455 0.012 C26 C30
DFIX_PM4 1.397 0.011 C26 C27
DFIX_PM4 1.386 0.010 C27 C28
DFIX_PM4 1.386 0.010 C28 C29
DFIX_PM4 1.509 0.011 C28 C28A
```

```
DFIX_PM4 1.272 0.007 C30 N31
DFIX_PM4 1.316 0.030 N31 O32
DFIX_PM4 1.325 0.030 N34 O33
DFIX_PM4 1.272 0.007 C35 N34
DFIX_PM4 1.455 0.012 C35 C36
DFIX_PM4 1.397 0.011 C36 C41
DFIX_PM4 1.431 0.013 C36 C37
DFIX_PM4 1.431 0.013 C37 C38
DFIX_PM4 1.267 0.029 C37 O37A
DFIX_PM4 1.397 0.011 C38 C39
DFIX_PM4 1.455 0.012 C38 C42
DFIX_PM4 1.386 0.010 C39 C40
DFIX_PM4 1.509 0.011 C40 C40A
DFIX_PM4 1.386 0.010 C40 C41
DFIX_PM4 1.272 0.007 C42 N43
DFIX_PM4 1.324 0.030 N43 O44
DFIX_PM4 1.398 0.009 C45 C50
DFIX_PM4 1.398 0.009 C45 C46
DFIX_PM4 1.381 0.012 C46 C47
DFIX_PM4 1.333 0.011 C47 N48
DFIX_PM4 1.333 0.011 C49 N48
DFIX_PM4 1.381 0.012 C49 C50
DANG_PM4 2.622 0.019 B7 C2
DANG_PM4 2.362 0.018 C2 C6
DANG_PM4 2.622 0.019 B7 C6
DANG_PM4 2.421 0.014 C1 C3
DANG_PM4 2.394 0.018 C2 N4
DANG_PM4 2.268 0.019 C3 C5
DANG_PM4 2.394 0.018 C6 N4
DANG_PM4 2.421 0.014 C1 C5
DANG_PM4 2.364 0.054 C1 O33
DANG_PM4 2.335 0.055 C1 O8
DANG_PM4 2.395 0.053 O33 O8
DANG_PM4 2.348 0.055 C1 O21
DANG_PM4 2.396 0.053 O21 O33
DANG_PM4 2.392 0.054 O21 O8
DANG_PM4 2.410 0.051 B7 N9
DANG_PM4 2.151 0.046 C10 O8
DANG_PM4 2.373 0.019 C11 N9
DANG_PM4 2.497 0.022 C10 C12
DANG_PM4 2.466 0.020 C10 C16
DANG_PM4 2.465 0.019 C12 C16
DANG_PM4 2.389 0.044 C11 C13
DANG_PM4 2.368 0.030 C11 O12A
DANG_PM4 2.368 0.030 C13 O12A
DANG_PM4 2.497 0.022 C12 C17
DANG_PM4 2.465 0.019 C12 C14
DANG_PM4 2.466 0.020 C14 C17
DANG_PM4 2.435 0.018 C13 C15
DANG_PM4 2.520 0.016 C14 C15A
DANG_PM4 2.377 0.016 C14 C16
DANG_PM4 2.520 0.016 C15A C16
DANG_PM4 2.435 0.018 C11 C15
DANG_PM4 2.373 0.019 C13 N18
DANG_PM4 2.157 0.046 C17 O19
DANG_PM4 2.410 0.051 B20 N18
DANG_PM4 2.410 0.053 O19 O44
DANG_PM4 2.348 0.055 C45 O19
DANG_PM4 2.377 0.054 C45 O44
DANG_PM4 2.380 0.054 O19 O32
```

```
DANG_PM4 2.403 0.053 O32 O44
DANG_PM4 2.346 0.055 C45 O32
DANG_PM4 2.378 0.051 B7 N22
DANG_PM4 2.169 0.045 C23 O21
DANG_PM4 2.373 0.019 C24 N22
DANG_PM4 2.497 0.022 C23 C25
DANG_PM4 2.466 0.020 C23 C29
DANG_PM4 2.465 0.019 C25 C29
DANG_PM4 2.368 0.030 C24 O25A
DANG_PM4 2.414 0.043 C24 C26
DANG_PM4 2.368 0.030 C26 O25A
DANG_PM4 2.497 0.022 C25 C30
DANG_PM4 2.465 0.019 C25 C27
DANG_PM4 2.466 0.020 C27 C30
DANG_PM4 2.435 0.018 C26 C28
DANG_PM4 2.377 0.016 C27 C29
DANG_PM4 2.520 0.016 C27 C28A
DANG_PM4 2.520 0.016 C28A C29
DANG_PM4 2.435 0.018 C24 C28
DANG_PM4 2.373 0.019 C26 N31
DANG_PM4 2.167 0.045 C30 O32
DANG_PM4 2.357 0.051 B20 N31
DANG_PM4 2.399 0.051 B7 N34
DANG_PM4 2.144 0.046 C35 O33
DANG_PM4 2.373 0.019 C36 N34
DANG_PM4 2.466 0.020 C35 C41
DANG_PM4 2.497 0.022 C35 C37
DANG_PM4 2.465 0.019 C37 C41
DANG_PM4 2.389 0.044 C36 C38
DANG_PM4 2.368 0.030 C36 O37A
DANG_PM4 2.368 0.030 C38 O37A
DANG_PM4 2.465 0.019 C37 C39
DANG_PM4 2.497 0.022 C37 C42
DANG_PM4 2.466 0.020 C39 C42
DANG_PM4 2.435 0.018 C38 C40
DANG_PM4 2.520 0.016 C39 C40A
DANG_PM4 2.377 0.016 C39 C41
DANG_PM4 2.520 0.016 C40A C41
DANG_PM4 2.435 0.018 C36 C40
DANG_PM4 2.373 0.019 C38 N43
DANG_PM4 2.149 0.046 C42 O44
DANG_PM4 2.395 0.051 B20 N43
DANG_PM4 2.622 0.019 B20 C50
DANG_PM4 2.622 0.019 B20 C46
DANG_PM4 2.362 0.018 C46 C50
DANG_PM4 2.421 0.014 C45 C47
DANG_PM4 2.394 0.018 C46 N48
DANG_PM4 2.268 0.019 C47 C49
DANG_PM4 2.394 0.018 C50 N48
DANG_PM4 2.421 0.014 C45 C49
FLAT_PM4 C1 C2 C6 B7
FLAT_PM4 C11 C10 C12 C16
FLAT_PM4 C13 C12 C14 C17
FLAT_PM4 C15 C14 C16 C15A
FLAT_PM4 C24 C23 C25 C29
FLAT_PM4 C26 C25 C27 C30
FLAT_PM4 C28 C27 C29 C28A
FLAT_PM4 C36 C35 C37 C41
FLAT_PM4 C38 C37 C39 C42
FLAT_PM4 C40 C39 C41 C40A
```

```
FLAT_PM4 C45 B20 C46 C50
FLAT_PM4 N9 C10 C11 C12
FLAT_PM4 C12 C13 C17 N18
FLAT_PM4 N22 C23 C24 C25
FLAT_PM4 C25 C26 C30 N31
FLAT_PM4 N34 C35 C36 C37
FLAT_PM4 C37 C38 C42 N43
FLAT_PM4 C12 C11 C13 O12A
FLAT_PM4 C25 C24 C26 O25A
FLAT_PM4 C37 C36 C38 O37A
FLAT_PM4 B7 O8 N9 C10
FLAT_PM4 O8 N9 C10 C11
FLAT_PM4 C10 C11 C16 C15
FLAT_PM4 C12 C13 C14 C15
FLAT_PM4 C13 C14 C15 C15A
FLAT_PM4 C15A C15 C16 C11
FLAT_PM4 C13 C17 N18 O19
FLAT_PM4 C17 N18 O19 B20
FLAT_PM4 B7 O21 N22 C23
FLAT_PM4 O21 N22 C23 C24
FLAT_PM4 C26 C30 N31 O32
FLAT_PM4 C30 N31 O32 B20
FLAT_PM4 B7 O33 N34 C35
FLAT_PM4 O33 N34 C35 C36
FLAT_PM4 C35 C36 C41 C40
FLAT_PM4 C37 C38 C39 C40
FLAT_PM4 C38 C42 N43 O44
FLAT_PM4 C42 N43 O44 B20
FLAT_PM4 C1 C2 C3 N4
FLAT_PM4 C2 C3 N4 C5
FLAT_PM4 C3 N4 C5 C6
FLAT_PM4 N4 C5 C6 C1
FLAT_PM4 C5 C6 C1 C2
FLAT_PM4 C6 C1 C2 C3
FLAT_PM4 C24 C25 C26 C27
FLAT_PM4 C25 C26 C27 C28
FLAT_PM4 C26 C27 C28 C29
FLAT_PM4 C27 C28 C29 C24
FLAT_PM4 C28 C29 C24 C25
FLAT_PM4 C29 C24 C25 C26
FLAT_PM4 C45 C46 C47 N48
FLAT_PM4 C46 C47 N48 C49
FLAT_PM4 C47 N48 C49 C50
FLAT_PM4 N48 C49 C50 C45
FLAT_PM4 C49 C50 C45 C46
FLAT_PM4 C50 C45 C46 C47
```

Program Structure

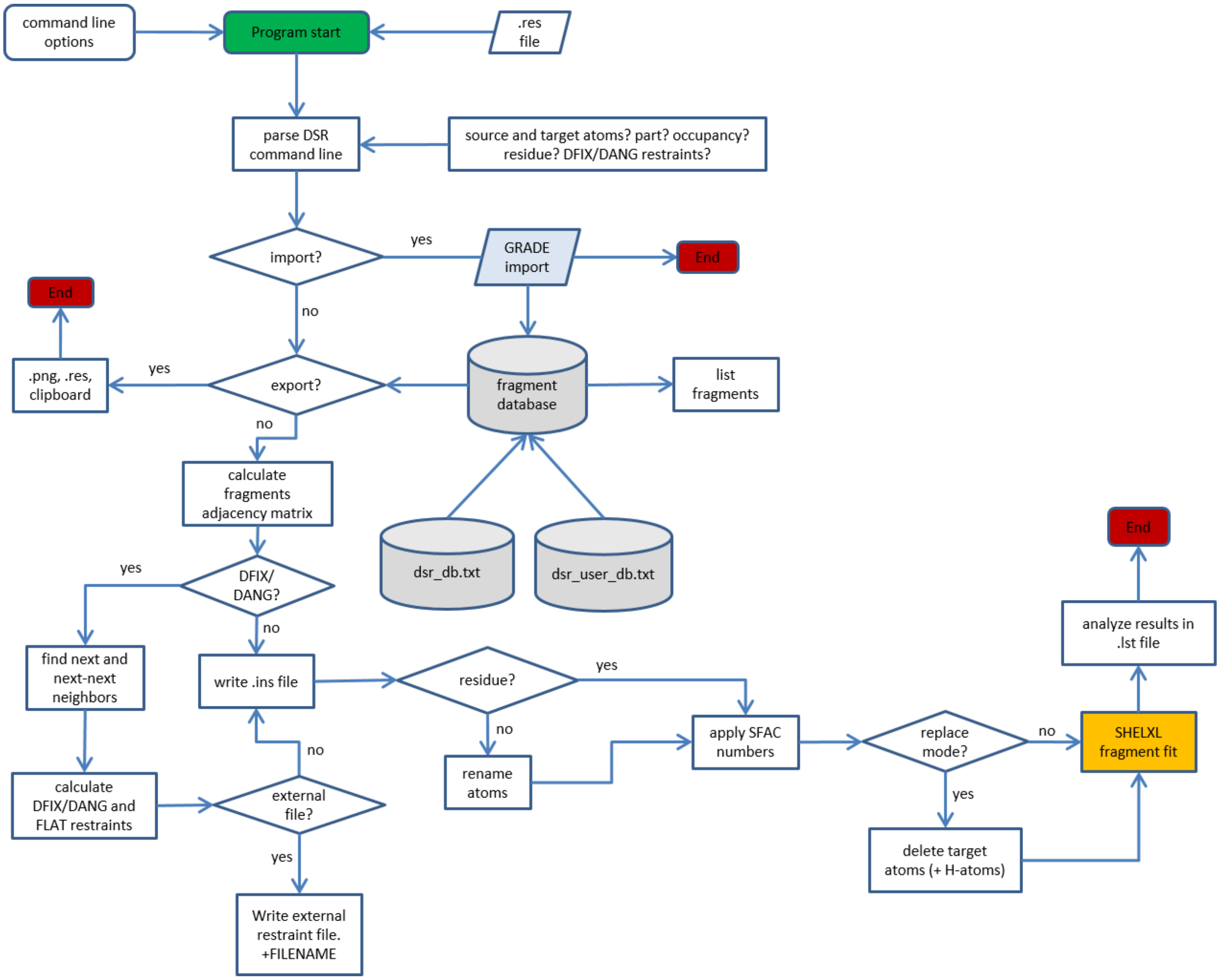

Command Line Syntax

Following options are available in the Windows or Unix command line to control the behavior of DSR:
usage: dsr [-h] [-r "res file"] [-re "res file"] [-e "fragment"] [-c "fragment"] [-t] [-i "tgz file"] [-l] [-n]
optional arguments:

–h, --help show a help message and exit.

–r "res file" res file with DSR command. Usually this option is used to process the SHELXL file with DSR.

–re "res file" Same as "-r", but a file called dsr_class_name.dfx or dsr_class_number_name.dfx is written which includes the restraints for the fragment for the .res file "name" in the residue "class" and "number".

–e "fragment" Exports a fragment from the database to the file [fragment].res. It includes the minimal requirements to view the fragment in a 3D molecule viewer. If a PLATON executable and ImageMagic installation is in the system path, it also creates a .png-picture of the molecule.

–c "fragment" Exports the fragment to the clipboard with cartesian coordinates. This fragment can for example be used for modelling in the program Olex2.

–t Inverts the current fragment. Available for fragment fit, import and export.

–i "GRADE file" Imports a molecular fragment from .tgz file of the Grade server http://grade.globalphasing.org/ into the dsr_usr_db.txt.

–l Displays all fragments in the database with the line numbers where they occur

–s "string" search the database for given string

–n Only transfers the fragment. Automatic fragment fit after the fragment transfer disabled.

Database

Three typical examples of database entries are listed below. The lines starting with "rem Src:" and "rem Name:" are not required but provide additional information for each database entry in the supplied main database. The name in the "rem Name:" line also appears in the listing of the "dsr –l" output and can be searched with the –s option.

```
<OC(CF3)3>
rem Name: Nonafluoro-tert-butoxy, [(CF3)3CO]-
rem Src: TURBOMOLE V6.0  B-P86/def-SV(P)
RESI CCF3
SADI 0.02 C1 C2 C1 C3 C1 C4
SADI 0.02 F1 C2 F2 C2 F3 C2 F4 C3 F5 C3 F6 C3 F7 C4 F8 C4 F9 C4
SADI 0.04 C2 C3 C3 C4 C2 C4
SADI 0.04 O1 C2 O1 C3 O1 C4
SADI 0.04 F1 F2 F2 F3 F3 F1 F4 F5 F5 F6 F6 F4 F7 F8 F8 F9 F9 F7
# The soft SADI restraint allows the CF3 group to tilt a little:
SADI 0.1 F1 C1 F2 C1 F3 C1 F4 C1 F5 C1 F6 C1 F7 C1 F8 C1 F9 C1
SIMU O1 > F9
RIGU O1 > F9
FRAG 17  1  1  1  90  90  90
O1   1      -0.01453        1.66590        0.10966
C1   1      -0.00146        0.26814        0.06351
C2   1      -1.13341       -0.23247       -0.90730
F1   1      -2.34661       -0.11273       -0.34544
F2   1      -0.96254       -1.50665       -1.29080
F3   1      -1.12263        0.55028       -2.01763
C3   1       1.40566       -0.23179       -0.43131
F4   1       2.38529        0.42340        0.20561
F5   1       1.53256        0.03843       -1.75538
F6   1       1.57833       -1.55153       -0.25035
C4   1      -0.27813       -0.21605        1.52795
F7   1       0.80602       -0.03759        2.30431
F8   1      -0.58910       -1.52859        1.53460
F9   1      -1.29323        0.46963        2.06735
</OC(CF3)3>

<THF>
rem Src: Gaussian 03 B3LYP/6-311++G(d,p) Opt
Rem Name: Tetrahydrofurane, thf
RESI THF
SADI 0.02  C1 C2  C3 C4
SADI 0.02  O1 C1  O1 C4
SADI 0.04  O1 C2  O1 C3
SADI 0.04  C2 C4  C1 C3
SIMU O1 > C4
RIGU O1 > C4
FRAG 17 1  1  1  90  90  90
O1    8      -0.000002   -1.251859   -0.000006
C1    6      -1.169782   -0.428968   -0.130825
C2    6      -0.734164    0.995903    0.225049
C3    6       0.734171    0.995898   -0.225052
C4    6       1.169778   -0.428974    0.130832
</THF>

<Benzene>
REM Name: Benzene, Benzol, C6H6
REM SRC: UGEDEQ
RESI BENZ
SADI 0.02 C1 C2 C2 C3 C3 C4 C4 C5 C5 C6 C6 C1
SADI 0.04 C1 C5 C1 C5 C4 C2 C4 C6 C6 C2 C5 C3
FLAT C1 > C6
SIMU C1 > C6
RIGU C1 > C6
FRAG  17  11.099 9.791 26.324 90 90 90
C1      1  0.880000 -0.330900  0.267190  1.000000 0.05000
C2      1  0.786200 -0.377700  0.238080  1.000000 0.05000
C3      1  0.760600 -0.318400  0.192570  1.000000 0.05000
```

```
C4      1  0.829200 -0.212200  0.175520  1.000000 0.05000
C5      1  0.923200 -0.164400  0.204000  1.000000 0.05000
C6      1  0.948800 -0.223200  0.249920  1.000000 0.05000
</Benzene>
```

Fragment Import from GRADE Web Server

DSR can import molecular fragments directly from the .tgz file generated by Grade Web Server of Global Phasing Ltd. (Smart & Womack, 2014) from which it reads the .dfix file for the restraints and runtime information, the .pdb file for the coordinates as well as comments and the name. All these information are then stored in the user database "dsr_user_db.txt". As it is essential that the fragment has proper names before being imported to the DSR user database, appropriate compound and residue names should already be provided to the Grade Web Server. Additionally, the output of SHELX restraints (e.g. non-hydrogen atoms only) has to be activated.

Fragment Export

DSR has several options to export molecular fragments. The option –e exports a .res file to be viewed in a regular structure editor. If PLATON (Spek, 2009) is found in the system PATH variable, an additional postscript picture (.ps) of the molecule is written to the hard disk. If also ImageMagic (*ImageMagick*, 2014) is installed, the .ps file is also converted to a .png file and deleted after successful conversion.

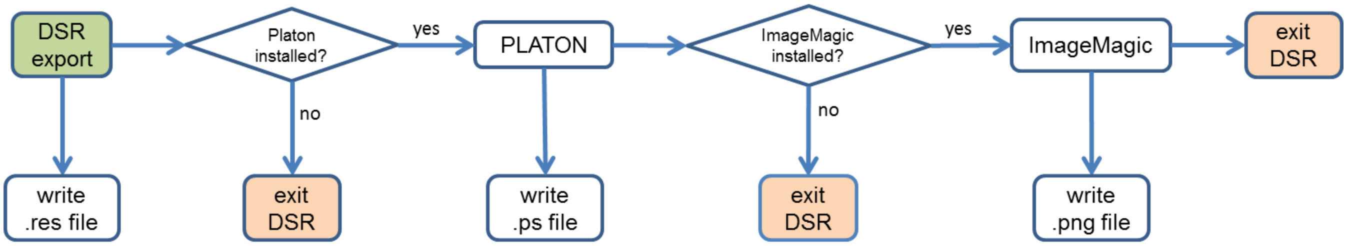

The option –c exports the fragment to the clip board. This clipboard entry can then be used for the *match* functionality in the program Olex2 (Guzei, 2014; Dolomanov *et al.*, 2009).

Example of Toluene fragment exported to the clip board:

```
FRAG
C1   1     1.7810   7.1491  12.0042
C2   1     2.2009   8.3068  11.1376
C3   1     1.2689   9.0217  10.3903
C4   1     1.6422  10.0777   9.5884
C5   1     2.9808  10.4443   9.5173
C6   1     3.9205   9.7497  10.2541
C7   1     3.5389   8.6909  11.0530
FEND
```

Automatic Restraints

For molecules with low occupancy or in a diffuse environment, the use of explicit distance restraints (DFIX, DANG) can be more powerful than similarity restraints (SADI). Therefore, the DSR command DFIX ignores all restraints in the database and generates DFIX/DANG/FLAT restraints for all 1,2- and 1,3-bond distances and planar bonding environments occurring in the fragment and it's neighboring atoms. This implies that the bond distances of the database fragment should be of sufficient accuracy to result in correct bond restraints. Especially the bond distances to other moieties are only as accurate as the initial fragment fit. Therefore, DSR warns about a possible inaccurate restraint in this case. Nevertheless, the main purpose of the automatic restraints is to get a stable starting model which needs to be further optimized in the subsequent refinement.
DSR generates the DFIX/DANG restraints automatically during the fragment fit by analyzing the fitted fragment and its surrounding atoms in the connectivity table for their next and second-next neighbors with a combination of the connectivity list from the SHELXL .lst file (1,2-distances) and by graph theoretical concepts via the NetworkX software library

(Hagberg *et al.*, 2013). An adjacency matrix of the connected atoms is generated with the atomic distances as weight. This matrix allows to obtain the 1,3-distances and finds rings, which are inside the fitted fragment and its neighboring atoms. The planarity of rings is analyzed similar to the FLAT restraint in SHELX by the volume of the tetrahedron spanned by groups of four atoms from the ring. FLAT restraints are generated for them accordingly.
For example, the distance restraints generated for the example molecule of the main paper are as follows:

```
DFIX 1.3432  F6_4   C3_4
DFIX 1.3489  F8_4   C4_4
DFIX 1.3453  C4_4   F7_4
DFIX 1.5670  C4_4   C1_4
DFIX 1.3385  C4_4   F9_4
DFIX 1.3424  F1_4   C2_4
DFIX 1.3586  F3_4   C2_4
DFIX 1.3573  C3_4   F5_4
DFIX 1.5732  C3_4   C1_4
DFIX 1.3397  C3_4   F4_4
DFIX 1.3986  C1_4   O1_4
DFIX 1.5731  C1_4   C2_4
DFIX 1.6857  O1_4   AL1_0
DFIX 1.3414  F2_4   C2_4
DANG 2.4313  O1_4   C3_4
DANG 2.3713  O1_4   C4_4
DANG 2.4271  O1_4   C2_4
DANG 2.9837  C1_4   AL1_0
DANG 2.4301  C1_4   F6_4
DANG 2.3905  C1_4   F5_4
DANG 2.3960  C1_4   F4_4
DANG 2.3954  C1_4   F8_4
DANG 2.4014  C1_4   F7_4
DANG 2.3925  C1_4   F9_4
DANG 2.4305  C1_4   F2_4
DANG 2.4108  C1_4   F1_4
DANG 2.3808  C1_4   F3_4
DANG 2.5834  C2_4   C3_4
DANG 2.5812  C2_4   C4_4
DANG 2.1800  F1_4   F2_4
DANG 2.1759  F1_4   F3_4
DANG 2.1874  F2_4   F3_4
DANG 2.5834  C3_4   C4_4
DANG 2.1816  F4_4   F6_4
DANG 2.1728  F4_4   F5_4
DANG 2.1899  F5_4   F6_4
DANG 2.1822  F7_4   F8_4
DANG 2.1726  F7_4   F9_4
DANG 2.1846  F8_4   F9_4
```

The distance restraints from the fitted fragment to the main molecule e.g. DFIX 1.6857 O1_4 AL1_0 should be carefully revised. They are only as accurate as the fit of the fragment.

Naming Scheme

Without using residues during a fragment fit with DSR the program defines a unique set of atom names to avoid clashes with the already existing atom names. Therefore, DSR numbers each element from 1 to the number of atoms of this element, than compares the names with the existing atom names. If an atom name already exists, a character from A to Z is appended to the name until every atom name is unique.

Disorder in the CSD

Table 1 Number of structures including disordered atoms in the CSD ranked by journal. Search preformed in March 2014.

Journal	No. of disordered structures in the CSD
Inorg. Chem.	18164
Acta Crystallogr.	10521
Organometallics	10349
J. Am. Chem. Soc.	10127
Dalton Trans.	8246
Chem.Commun.	6782
Angew. Chem.	5702
Chem.-Eur. J.	5308
Inorg. Chim. Acta	5294
J. Chem. Soc.	5209

Example res-file before DSR

```
TITL p21c in P2(1)/c
CELL 0.71073 10.5086 20.9035 20.5072 90 94.13 90
ZERR 4 0.0003 0.0005 0.0005 0 0.001 0
LATT 1
SYMM -X,0.5+Y,0.5-Z
SFAC  C  H  O  F  Al  Ga
UNIT  1  2  3  4  5  6
LIST 6 ! automatically inserted. Change 6 to 4 for CHECKCIF!!

L.S.  0
PLAN  20
TEMP -173.18
BOND $H
fmap 2

WGHT    0.049400    0.393100
FVAR       0.08470   0.48340   0.55590
GA1   6    0.639418    0.561703    0.237772    11.00000    0.02258    0.02440 =
         0.02432    0.00008   -0.00145    0.00127
AL1   5    0.064158    0.260145    0.478645    11.00000    0.01537    0.01791 =
         0.01248    0.00026   -0.00269    0.00132
C1    1    0.721483    0.447407    0.167507    11.00000    0.02925    0.02416 =
         0.02757    0.00048   -0.00135    0.01034
C2    1    0.838817    0.474526    0.190081    11.00000    0.02664    0.02750 =
         0.02625    0.00135    0.00685    0.00578
C3    1    0.875251    0.478410    0.256955    11.00000    0.02154    0.02507 =
         0.03766    0.00476   -0.01051    0.00493
C4    1    0.789290    0.456520    0.301616    11.00000    0.03898    0.02307 =
```

```
          0.02337    0.00026   -0.00678    0.01112
C5    1    0.674054    0.430194    0.280727    11.00000    0.02406    0.01783 =
          0.03400    0.00389    0.00453    0.00367
C6    1    0.641561    0.425245    0.213299    11.00000    0.02446    0.01826 =
          0.03459   -0.00035   -0.00290   -0.00150
C7    1    0.685281    0.443804    0.095051    11.00000    0.05870    0.03446 =
          0.02786   -0.01108   -0.00364    0.00426
C8    1    1.004216    0.505479    0.281521    11.00000    0.02767    0.05745 =
          0.05216    0.00998   -0.01135   -0.00116
C9    1    0.588465    0.401681    0.329036    11.00000    0.04007    0.04636 =
          0.03676    0.00855    0.00751   -0.00170
C10   1    0.399926    0.588094    0.161257    11.00000    0.01897    0.02882 =
          0.01756   -0.00178   -0.00575    0.00263
C11   1    0.443959    0.649581    0.176207    11.00000    0.01984    0.02394 =
          0.02167    0.00432   -0.00109    0.00413
C12   1    0.454920    0.672343    0.241043    11.00000    0.01482    0.02767 =
          0.03155   -0.00377   -0.00368    0.00311
C13   1    0.425895    0.630971    0.290285    11.00000    0.01698    0.03346 =
          0.02421   -0.00848   -0.00087    0.00502
C14   1    0.380809    0.569642    0.277486    11.00000    0.01555    0.03193 =
          0.02070   -0.00127   -0.00368    0.00481
C15   1    0.369916    0.548581    0.213715    11.00000    0.02056    0.02385 =
          0.03115   -0.00483   -0.00132   -0.00159
C16   1    0.385894    0.565447    0.092123    11.00000    0.05037    0.03031 =
          0.02353    0.00318   -0.00502    0.00546
C17   1    0.493624    0.740392    0.255361    11.00000    0.03899    0.02492 =
          0.04233   -0.00684    0.00093   -0.00388
C18   1    0.341537    0.527543    0.332385    11.00000    0.02457    0.04511 =
          0.03136    0.00233    0.00174   -0.00756
RESI 1 CF3
PART 1 21
O1    3    0.156692    0.209642    0.530001    21.00000    0.02315    0.02219 =
          0.01893    0.00227   -0.00526    0.00515
C1    1    0.197695    0.149686    0.544131    21.00000    0.02311    0.01933 =
          0.02257    0.00291   -0.00585    0.00520
C2    1    0.282991    0.125315    0.491233    21.00000    0.03936    0.03109 =
          0.02323    0.00341   -0.00131    0.01347
F1    4    0.360748    0.075978    0.512602    21.00000    0.03664    0.03565 =
          0.03483    0.00716    0.00383    0.01729
F2    4    0.359755    0.171939    0.471788    21.00000    0.04109    0.03438 =
          0.03281   -0.00015    0.01321    0.01417
F3    4    0.210835    0.104067    0.437922    21.00000    0.07243    0.03058 =
          0.03216   -0.01057   -0.01708    0.03014
C3    1    0.086991    0.103824    0.549299    21.00000    0.02692    0.03316 =
          0.05474    0.01002   -0.00086    0.00109
F4    4    0.120410    0.041250    0.544284    21.00000    0.03266    0.03244 =
          0.07659    0.01314    0.00589   -0.00254
F5    4   -0.004747    0.113714    0.499154    21.00000    0.02871    0.04631 =
          0.06699   -0.00018   -0.00756    0.00283
F6    4    0.032053    0.111727    0.606598    21.00000    0.04342    0.04534 =
          0.06816    0.01912    0.01300    0.00142
C4    1    0.278833    0.152099    0.609841    21.00000    0.02997    0.02802 =
          0.02149    0.00544   -0.00704    0.00570
F7    4    0.217441    0.187353    0.652825    21.00000    0.03887    0.04131 =
          0.02281    0.00659   -0.00204    0.00654
F8    4    0.297811    0.093412    0.637836    21.00000    0.05603    0.03390 =
          0.03278    0.01228   -0.01698    0.00691
F9    4    0.395366    0.177026    0.601546    21.00000    0.03231    0.03248 =
          0.03649   -0.00522   -0.01212    0.00157
RESI 0
```

```
PART 0

rem dsr put oc(cf3)3 with o1 c1 c2 c3 on O1_1 C1_1 q15 q14 part 2 -21

RESI 3 CF3
PART 1 31
O1    3    0.081800    0.235411    0.399962    31.00000    0.02269
C1    1    0.033372    0.232537    0.337248    31.00000    0.02263
C2    1    0.146121    0.230044    0.293483    31.00000    0.07563
F1    4    0.113536    0.207456    0.233219    31.00000    0.04423
F2    4    0.245556    0.200608    0.318034    31.00000    0.04443
F3    4    0.177383    0.298455    0.290893    31.00000    0.06590
C3    1   -0.047007    0.289792    0.317142    31.00000    0.06657
F4    4   -0.015523    0.342850    0.348530    31.00000    0.04231
F5    4   -0.064007    0.300531    0.253531    31.00000    0.03919
F6    4   -0.174400    0.269329    0.340816    31.00000    0.06549
C4    1   -0.042812    0.170935    0.325702    31.00000    0.06900
F7    4   -0.111534    0.156135    0.374179    31.00000    0.04207
F8    4   -0.120582    0.172784    0.270319    31.00000    0.05058
F9    4    0.058836    0.122805    0.319484    31.00000    0.06663
RESI 0
PART 0

RESI 2 CF3
O1    3    0.120080    0.336659    0.494426    11.00000    0.01445    0.02176 =
         0.02347   -0.00009   -0.00214   -0.00291
C1    1    0.223789    0.375512    0.504147    11.00000    0.01922    0.02446 =
         0.01675   -0.00034   -0.00606    0.00209
C2    1    0.281319    0.368769    0.575106    11.00000    0.02786    0.03396 =
         0.02473   -0.00516   -0.00826   -0.00253
F1    4    0.352077    0.314955    0.582945    11.00000    0.03615    0.03269 =
         0.04176    0.00669   -0.02389    0.00497
F2    4    0.191896    0.365437    0.617058    11.00000    0.04052    0.05331 =
         0.01801    0.00126    0.00065   -0.01022
F3    4    0.358359    0.417667    0.594043    11.00000    0.03351    0.03715 =
         0.03469   -0.00423   -0.01418   -0.01030
C3    1    0.180032    0.444507    0.492855    11.00000    0.02563    0.02125 =
         0.02837    0.00266    0.00070   -0.00523
F4    4    0.116221    0.466033    0.542783    11.00000    0.03319    0.02567 =
         0.03803   -0.00809    0.00266    0.00136
F5    4    0.276593    0.484828    0.485871    11.00000    0.02781    0.02726 =
         0.04871    0.00319    0.00259   -0.00680
F6    4    0.100759    0.449528    0.438703    11.00000    0.03898    0.03388 =
         0.03322    0.00908   -0.00968    0.00768
C4    1    0.324256    0.359363    0.456165    11.00000    0.01865    0.02847 =
         0.03315    0.00171    0.00208   -0.00016
F7    4    0.439198    0.385328    0.473759    11.00000    0.01729    0.04404 =
         0.05057   -0.00420    0.00175   -0.00678
F8    4    0.290860    0.379953    0.395614    11.00000    0.03767    0.04288 =
         0.02398    0.00162    0.00874   -0.00142
F9    4    0.342682    0.296329    0.451511    11.00000    0.02300    0.02615 =
         0.05130   -0.01040    0.00470    0.00386
RESI 0
RESI 4 CF3
O1    3   -0.097841    0.261274    0.489484    11.00000    0.01763    0.02418 =
         0.01555   -0.00406    0.00248    0.00104
C1    1   -0.186843    0.282708    0.526803    11.00000    0.01701    0.01847 =
         0.01798    0.00022   -0.00212   -0.00417
C2    1   -0.155278    0.264593    0.600644    11.00000    0.02003    0.03844 =
         0.02341   -0.00570   -0.00223    0.00368
```

```
F1    4   -0.031112    0.275566    0.616775    11.00000    0.02552    0.05113 =
         0.02260   -0.00420   -0.00727    0.00468
F2    4   -0.175472    0.202321    0.610260    11.00000    0.03904    0.03977 =
         0.03003    0.01650    0.00263    0.00387
F3    4   -0.222817    0.296838    0.641346    11.00000    0.03175    0.06390 =
         0.01794   -0.00770    0.00409    0.01074
C3    1   -0.200650    0.357301    0.522420    11.00000    0.02037    0.03111 =
         0.02907   -0.00643   -0.00278    0.00165
F4    4   -0.110636    0.386539    0.560147    11.00000    0.02804    0.02847 =
         0.04540   -0.01592   -0.00126   -0.00172
F5    4   -0.189167    0.376459    0.461529    11.00000    0.04719    0.02557 =
         0.03265    0.00720    0.00266    0.00222
F6    4   -0.313803    0.377149    0.540550    11.00000    0.02245    0.03301 =
         0.04168   -0.00621    0.00099    0.00914
C4    1   -0.316216    0.251064    0.503877    11.00000    0.02382    0.03163 =
         0.02487    0.00288   -0.00174    0.00015
F7    4   -0.362398    0.278516    0.447770    11.00000    0.02302    0.04023 =
         0.02897    0.00131   -0.01216    0.00374
F8    4   -0.404458    0.257464    0.546968    11.00000    0.01762    0.05015 =
         0.03179   -0.00160    0.00590   -0.00354
F9    4   -0.301403    0.189114    0.492650    11.00000    0.02542    0.02124 =
         0.04111   -0.00397   -0.00273   -0.00460
RESI 0

HKLF 4

REM  p21c in P2(1)/c
REM R1 =  0.2134 for    7085 Fo > 4sig(Fo)  and  0.2583 for all   10786 data
REM    617 parameters refined using      0 restraints

END

WGHT      0.2000      0.0000

REM Highest difference peak  6.621,  deepest hole -1.137,  1-sigma level  0.428
Q1    1  -0.0286  0.1132  0.5221  11.00000  0.05    6.62
Q2    1   0.2495  0.1920  0.6498  11.00000  0.05    6.56
Q3    1   0.1082  0.0834  0.4532  11.00000  0.05    5.74
Q4    1   0.0777  0.1305  0.6436  11.00000  0.05    5.68
Q5    1   0.3212  0.1569  0.4585  11.00000  0.05    5.58
Q6    1   0.4076  0.1667  0.5561  11.00000  0.05    5.37
Q7    1   0.2686  0.0899  0.6398  11.00000  0.05    5.15
Q8    1  -0.1946  0.2406  0.3487  11.00000  0.05    4.39
Q9    1   0.1246  0.3285  0.2932  11.00000  0.05    4.17
Q10   1   0.1159  0.1320  0.3115  11.00000  0.05    4.09
Q11   1   0.3175  0.1365  0.5196  11.00000  0.05    3.79
Q12   1   0.2101  0.2873  0.2901  11.00000  0.05    3.60
Q13   1  -0.1637  0.2879  0.3375  11.00000  0.05    3.37
Q14   1   0.0901  0.0918  0.5162  11.00000  0.05    3.24
Q15   1   0.1957  0.1390  0.6188  11.00000  0.05    3.18
Q16   1   0.0280  0.1138  0.3279  11.00000  0.05    3.06
Q17   1   0.1255  0.1937  0.2957  11.00000  0.05    2.84
Q18   1   0.0214  0.3063  0.3072  11.00000  0.05    2.76
Q19   1  -0.1058  0.2040  0.3332  11.00000  0.05    2.49
Q20   1   0.2469  0.5560  0.3511  11.00000  0.05    1.08
```

Example res-file before fragment fit

```
TITL p21c in P2(1)/c
CELL 0.71073 10.5086 20.9035 20.5072 90 94.13 90
ZERR 4 0.0003 0.0005 0.0005 0 0.001 0
LATT 1
SYMM -X,0.5+Y,0.5-Z
SFAC  C  H  O  F  Al  Ga
UNIT  1  2  3  4  5  6
LIST 6 ! automatically inserted. Change 6 to 4 for CHECKCIF!!

L.S.  0
PLAN  20
TEMP -173.18
BOND $H
fmap 2

WGHT    0.049400    0.393100
FVAR       0.08470   0.48340   0.55590

 The following is from DSR:
FRAG  17  1  1  1  90  90  90
O1    3     -0.014530      1.665900      0.109660
C1    1     -0.001460      0.268140      0.063510
C2    1     -1.133410     -0.232470     -0.907300
F1    4     -2.346610     -0.112730     -0.345440
F2    4     -0.962540     -1.506650     -1.290800
F3    4     -1.122630      0.550280     -2.017630
C3    1      1.405660     -0.231790     -0.431310
F4    4      2.385290      0.423400      0.205610
F5    4      1.532560      0.038430     -1.755380
F6    4      1.578330     -1.551530     -0.250350
C4    1     -0.278130     -0.216050      1.527950
F7    4      0.806020     -0.037590      2.304310
F8    4     -0.589100     -1.528590      1.534600
F9    4     -1.293230      0.469630      2.067350
FEND

GA1   6    0.639418    0.561703    0.237772    11.00000    0.02258    0.02440 =
         0.02432    0.00008   -0.00145    0.00127
AL1   5    0.064158    0.260145    0.478645    11.00000    0.01537    0.01791 =
         0.01248    0.00026   -0.00269    0.00132
C1    1    0.721483    0.447407    0.167507    11.00000    0.02925    0.02416 =
         0.02757    0.00048   -0.00135    0.01034
C2    1    0.838817    0.474526    0.190081    11.00000    0.02664    0.02750 =
         0.02625    0.00135    0.00685    0.00578
C3    1    0.875251    0.478410    0.256955    11.00000    0.02154    0.02507 =
         0.03766    0.00476   -0.01051    0.00493
C4    1    0.789290    0.456520    0.301616    11.00000    0.03898    0.02307 =
         0.02337    0.00026   -0.00678    0.01112
C5    1    0.674054    0.430194    0.280727    11.00000    0.02406    0.01783 =
         0.03400    0.00389    0.00453    0.00367
C6    1    0.641561    0.425245    0.213299    11.00000    0.02446    0.01826 =
         0.03459   -0.00035   -0.00290   -0.00150
C7    1    0.685281    0.443804    0.095051    11.00000    0.05870    0.03446 =
         0.02786   -0.01108   -0.00364    0.00426
C8    1    1.004216    0.505479    0.281521    11.00000    0.02767    0.05745 =
         0.05216    0.00998   -0.01135   -0.00116
C9    1    0.588465    0.401681    0.329036    11.00000    0.04007    0.04636 =
         0.03676    0.00855    0.00751   -0.00170
C10   1    0.399926    0.588094    0.161257    11.00000    0.01897    0.02882 =
```

```
          0.01756   -0.00178   -0.00575    0.00263
C11   1    0.443959    0.649581    0.176207    11.00000    0.01984    0.02394 =
          0.02167    0.00432   -0.00109    0.00413
C12   1    0.454920    0.672343    0.241043    11.00000    0.01482    0.02767 =
          0.03155   -0.00377   -0.00368    0.00311
C13   1    0.425895    0.630971    0.290285    11.00000    0.01698    0.03346 =
          0.02421   -0.00848   -0.00087    0.00502
C14   1    0.380809    0.569642    0.277486    11.00000    0.01555    0.03193 =
          0.02070   -0.00127   -0.00368    0.00481
C15   1    0.369916    0.548581    0.213715    11.00000    0.02056    0.02385 =
          0.03115   -0.00483   -0.00132   -0.00159
C16   1    0.385894    0.565447    0.092123    11.00000    0.05037    0.03031 =
          0.02353    0.00318   -0.00502    0.00546
C17   1    0.493624    0.740392    0.255361    11.00000    0.03899    0.02492 =
          0.04233   -0.00684    0.00093   -0.00388
C18   1    0.341537    0.527543    0.332385    11.00000    0.02457    0.04511 =
          0.03136    0.00233    0.00174   -0.00756
RESI 1 CF3
PART 1 21
O1    3    0.156692    0.209642    0.530001    21.00000    0.02315    0.02219 =
          0.01893    0.00227   -0.00526    0.00515
C1    1    0.197695    0.149686    0.544131    21.00000    0.02311    0.01933 =
          0.02257    0.00291   -0.00585    0.00520
C2    1    0.282991    0.125315    0.491233    21.00000    0.03936    0.03109 =
          0.02323    0.00341   -0.00131    0.01347
F1    4    0.360748    0.075978    0.512602    21.00000    0.03664    0.03565 =
          0.03483    0.00716    0.00383    0.01729
F2    4    0.359755    0.171939    0.471788    21.00000    0.04109    0.03438 =
          0.03281   -0.00015    0.01321    0.01417
F3    4    0.210835    0.104067    0.437922    21.00000    0.07243    0.03058 =
          0.03216   -0.01057   -0.01708    0.03014
C3    1    0.086991    0.103824    0.549299    21.00000    0.02692    0.03316 =
          0.05474    0.01002   -0.00086    0.00109
F4    4    0.120410    0.041250    0.544284    21.00000    0.03266    0.03244 =
          0.07659    0.01314    0.00589   -0.00254
F5    4   -0.004747    0.113714    0.499154    21.00000    0.02871    0.04631 =
          0.06699   -0.00018   -0.00756    0.00283
F6    4    0.032053    0.111727    0.606598    21.00000    0.04342    0.04534 =
          0.06816    0.01912    0.01300    0.00142
C4    1    0.278833    0.152099    0.609841    21.00000    0.02997    0.02802 =
          0.02149    0.00544   -0.00704    0.00570
F7    4    0.217441    0.187353    0.652825    21.00000    0.03887    0.04131 =
          0.02281    0.00659   -0.00204    0.00654
F8    4    0.297811    0.093412    0.637836    21.00000    0.05603    0.03390 =
          0.03278    0.01228   -0.01698    0.00691
F9    4    0.395366    0.177026    0.601546    21.00000    0.03231    0.03248 =
          0.03649   -0.00522   -0.01212    0.00157
RESI 0
PART 0


rem the following was inserted by DSR:
SADI 0.02 C1A C2A C1A C3A C1A C4A
SADI 0.02 F1A C2A F2A C2A F3A C2A F4A C3A F5A C3A F6A C3A F7A C4A F8A C4A F9A =
   C4A
SADI 0.04 C2A C3A C3A C4A C2A C4A
SADI 0.04 O1A C2A O1A C3A O1A C4A
SADI 0.04 F1A F2A F2A F3A F3A F1A F4A F5A F5A F6A F6A F4A F7A F8A F8A F9A F9A =
   F7A
SADI 0.1 F1A C1A F2A C1A F3A C1A F4A C1A F5A C1A F6A C1A F7A C1A F8A C1A F9A =
```

```
   C1A
SIMU O1A > F9A
RIGU O1A > F9A
PART 2
AFIX 179
O1A   3     0.156692     0.209642     0.530001   11.000   0.04
C1A   1     0.197695     0.149686     0.544131   11.000   0.04
C2A   1     0.195700     0.139000     0.618800   11.000   0.04
F1A   4     0.000000     0.000000     0.000000   11.000   0.04
F2A   4     0.000000     0.000000     0.000000   11.000   0.04
F3A   4     0.000000     0.000000     0.000000   11.000   0.04
C3A   1     0.090100     0.091800     0.516200   11.000   0.04
F4A   4     0.000000     0.000000     0.000000   11.000   0.04
F5A   4     0.000000     0.000000     0.000000   11.000   0.04
F6A   4     0.000000     0.000000     0.000000   11.000   0.04
C4A   1     0.000000     0.000000     0.000000   11.000   0.04
F7A   4     0.000000     0.000000     0.000000   11.000   0.04
F8A   4     0.000000     0.000000     0.000000   11.000   0.04
F9A   4     0.000000     0.000000     0.000000   11.000   0.04
AFIX 0
PART 0
rem The end of the DSR entry

rem rem dsr put oc(cf3)3 with o1 c1 c2 c3 on O1_1 C1_1 q15 q14 part 2 -21

RESI 3 CF3
PART 1 31
O1    3    0.081800    0.235411    0.399962    31.00000    0.02269
C1    1    0.033372    0.232537    0.337248    31.00000    0.02263
C2    1    0.146121    0.230044    0.293483    31.00000    0.07563
F1    4    0.113536    0.207456    0.233219    31.00000    0.04423
F2    4    0.245556    0.200608    0.318034    31.00000    0.04443
F3    4    0.177383    0.298455    0.290893    31.00000    0.06590
C3    1   -0.047007    0.289792    0.317142    31.00000    0.06657
F4    4   -0.015523    0.342850    0.348530    31.00000    0.04231
F5    4   -0.064007    0.300531    0.253531    31.00000    0.03919
F6    4   -0.174400    0.269329    0.340816    31.00000    0.06549
C4    1   -0.042812    0.170935    0.325702    31.00000    0.06900
F7    4   -0.111534    0.156135    0.374179    31.00000    0.04207
F8    4   -0.120582    0.172784    0.270319    31.00000    0.05058
F9    4    0.058836    0.122805    0.319484    31.00000    0.06663
RESI 0
PART 0

RESI 2 CF3
O1    3    0.120080    0.336659    0.494426    11.00000    0.01445    0.02176 =
         0.02347   -0.00009   -0.00214   -0.00291
C1    1    0.223789    0.375512    0.504147    11.00000    0.01922    0.02446 =
         0.01675   -0.00034   -0.00606    0.00209
C2    1    0.281319    0.368769    0.575106    11.00000    0.02786    0.03396 =
         0.02473   -0.00516   -0.00826   -0.00253
F1    4    0.352077    0.314955    0.582945    11.00000    0.03615    0.03269 =
         0.04176    0.00669   -0.02389    0.00497
F2    4    0.191896    0.365437    0.617058    11.00000    0.04052    0.05331 =
         0.01801    0.00126    0.00065   -0.01022
F3    4    0.358359    0.417667    0.594043    11.00000    0.03351    0.03715 =
         0.03469   -0.00423   -0.01418   -0.01030
C3    1    0.180032    0.444507    0.492855    11.00000    0.02563    0.02125 =
         0.02837    0.00266    0.00070   -0.00523
F4    4    0.116221    0.466033    0.542783    11.00000    0.03319    0.02567 =
```

```
         0.03803   -0.00809    0.00266    0.00136
F5    4    0.276593    0.484828    0.485871    11.00000    0.02781    0.02726 =
         0.04871    0.00319    0.00259   -0.00680
F6    4    0.100759    0.449528    0.438703    11.00000    0.03898    0.03388 =
         0.03322    0.00908   -0.00968    0.00768
C4    1    0.324256    0.359363    0.456165    11.00000    0.01865    0.02847 =
         0.03315    0.00171    0.00208   -0.00016
F7    4    0.439198    0.385328    0.473759    11.00000    0.01729    0.04404 =
         0.05057   -0.00420    0.00175   -0.00678
F8    4    0.290860    0.379953    0.395614    11.00000    0.03767    0.04288 =
         0.02398    0.00162    0.00874   -0.00142
F9    4    0.342682    0.296329    0.451511    11.00000    0.02300    0.02615 =
         0.05130   -0.01040    0.00470    0.00386
RESI 0
RESI 4 CF3
O1    3   -0.097841    0.261274    0.489484    11.00000    0.01763    0.02418 =
         0.01555   -0.00406    0.00248    0.00104
C1    1   -0.186843    0.282708    0.526803    11.00000    0.01701    0.01847 =
         0.01798    0.00022   -0.00212   -0.00417
C2    1   -0.155278    0.264593    0.600644    11.00000    0.02003    0.03844 =
         0.02341   -0.00570   -0.00223    0.00368
F1    4   -0.031112    0.275566    0.616775    11.00000    0.02552    0.05113 =
         0.02260   -0.00420   -0.00727    0.00468
F2    4   -0.175472    0.202321    0.610260    11.00000    0.03904    0.03977 =
         0.03003    0.01650    0.00263    0.00387
F3    4   -0.222817    0.296838    0.641346    11.00000    0.03175    0.06390 =
         0.01794   -0.00770    0.00409    0.01074
C3    1   -0.200650    0.357301    0.522420    11.00000    0.02037    0.03111 =
         0.02907   -0.00643   -0.00278    0.00165
F4    4   -0.110636    0.386539    0.560147    11.00000    0.02804    0.02847 =
         0.04540   -0.01592   -0.00126   -0.00172
F5    4   -0.189167    0.376459    0.461529    11.00000    0.04719    0.02557 =
         0.03265    0.00720    0.00266    0.00222
F6    4   -0.313803    0.377149    0.540550    11.00000    0.02245    0.03301 =
         0.04168   -0.00621    0.00099    0.00914
C4    1   -0.316216    0.251064    0.503877    11.00000    0.02382    0.03163 =
         0.02487    0.00288   -0.00174    0.00015
F7    4   -0.362398    0.278516    0.447770    11.00000    0.02302    0.04023 =
         0.02897    0.00131   -0.01216    0.00374
F8    4   -0.404458    0.257464    0.546968    11.00000    0.01762    0.05015 =
         0.03179   -0.00160    0.00590   -0.00354
F9    4   -0.301403    0.189114    0.492650    11.00000    0.02542    0.02124 =
         0.04111   -0.00397   -0.00273   -0.00460
RESI 0

HKLF 4

REM  p21c in P2(1)/c
REM R1 =  0.2134 for    7085 Fo > 4sig(Fo)  and  0.2583 for all   10786 data
REM    617 parameters refined using      0 restraints

END

WGHT      0.2000      0.0000

REM Highest difference peak  6.621,  deepest hole -1.137,  1-sigma level  0.428
Q1    1  -0.0286  0.1132  0.5221  11.00000  0.05    6.62
Q2    1   0.2495  0.1920  0.6498  11.00000  0.05    6.56
Q3    1   0.1082  0.0834  0.4532  11.00000  0.05    5.74
Q4    1   0.0777  0.1305  0.6436  11.00000  0.05    5.68
```

```
Q5    1   0.3212  0.1569  0.4585  11.00000  0.05    5.58
Q6    1   0.4076  0.1667  0.5561  11.00000  0.05    5.37
Q7    1   0.2686  0.0899  0.6398  11.00000  0.05    5.15
Q8    1  -0.1946  0.2406  0.3487  11.00000  0.05    4.39
Q9    1   0.1246  0.3285  0.2932  11.00000  0.05    4.17
Q10   1   0.1159  0.1320  0.3115  11.00000  0.05    4.09
Q11   1   0.3175  0.1365  0.5196  11.00000  0.05    3.79
Q12   1   0.2101  0.2873  0.2901  11.00000  0.05    3.60
Q13   1  -0.1637  0.2879  0.3375  11.00000  0.05    3.37
Q14   1   0.0901  0.0918  0.5162  11.00000  0.05    3.24
Q15   1   0.1957  0.1390  0.6188  11.00000  0.05    3.18
Q16   1   0.0280  0.1138  0.3279  11.00000  0.05    3.06
Q17   1   0.1255  0.1937  0.2957  11.00000  0.05    2.84
Q18   1   0.0214  0.3063  0.3072  11.00000  0.05    2.76
Q19   1  -0.1058  0.2040  0.3332  11.00000  0.05    2.49
Q20   1   0.2469  0.5560  0.3511  11.00000  0.05    1.08
```

Example res-file after DSR

```
TITL p21c in P2(1)/c
CELL 0.71073 10.5086 20.9035 20.5072 90 94.13 90
ZERR 4 0.0003 0.0005 0.0005 0 0.001 0
LATT 1
SYMM -X,0.5+Y,0.5-Z
SFAC  C  H  O  F  Al  Ga
UNIT  1  2  3  4  5  6
LIST 6 ! automatically inserted. Change 6 to 4 for CHECKCIF!!

L.S.  8
PLAN  20
TEMP -173.18
BOND $H
fmap 2

WGHT    0.049400    0.393100
FVAR       0.08470   0.48340   0.55590
GA1   6    0.639418    0.561703    0.237772    11.00000    0.02258    0.02440 =
         0.02432    0.00008   -0.00145    0.00127
AL1   5    0.064158    0.260145    0.478645    11.00000    0.01537    0.01791 =
         0.01248    0.00026   -0.00269    0.00132
C1    1    0.721483    0.447407    0.167507    11.00000    0.02925    0.02416 =
         0.02757    0.00048   -0.00135    0.01034
C2    1    0.838817    0.474526    0.190081    11.00000    0.02664    0.02750 =
         0.02625    0.00135    0.00685    0.00578
C3    1    0.875251    0.478410    0.256955    11.00000    0.02154    0.02507 =
         0.03766    0.00476   -0.01051    0.00493
C4    1    0.789290    0.456520    0.301616    11.00000    0.03898    0.02307 =
         0.02337    0.00026   -0.00678    0.01112
C5    1    0.674054    0.430194    0.280727    11.00000    0.02406    0.01783 =
         0.03400    0.00389    0.00453    0.00367
C6    1    0.641561    0.425245    0.213299    11.00000    0.02446    0.01826 =
         0.03459   -0.00035   -0.00290   -0.00150
C7    1    0.685281    0.443804    0.095051    11.00000    0.05870    0.03446 =
         0.02786   -0.01108   -0.00364    0.00426
C8    1    1.004216    0.505479    0.281521    11.00000    0.02767    0.05745 =
         0.05216    0.00998   -0.01135   -0.00116
C9    1    0.588465    0.401681    0.329036    11.00000    0.04007    0.04636 =
         0.03676    0.00855    0.00751   -0.00170
C10   1    0.399926    0.588094    0.161257    11.00000    0.01897    0.02882 =
```

```
          0.01756   -0.00178   -0.00575    0.00263
C11   1    0.443959    0.649581    0.176207    11.00000    0.01984    0.02394 =
          0.02167    0.00432   -0.00109    0.00413
C12   1    0.454920    0.672343    0.241043    11.00000    0.01482    0.02767 =
          0.03155   -0.00377   -0.00368    0.00311
C13   1    0.425895    0.630971    0.290285    11.00000    0.01698    0.03346 =
          0.02421   -0.00848   -0.00087    0.00502
C14   1    0.380809    0.569642    0.277486    11.00000    0.01555    0.03193 =
          0.02070   -0.00127   -0.00368    0.00481
C15   1    0.369916    0.548581    0.213715    11.00000    0.02056    0.02385 =
          0.03115   -0.00483   -0.00132   -0.00159
C16   1    0.385894    0.565447    0.092123    11.00000    0.05037    0.03031 =
          0.02353    0.00318   -0.00502    0.00546
C17   1    0.493624    0.740392    0.255361    11.00000    0.03899    0.02492 =
          0.04233   -0.00684    0.00093   -0.00388
C18   1    0.341537    0.527543    0.332385    11.00000    0.02457    0.04511 =
          0.03136    0.00233    0.00174   -0.00756
RESI 1 CF3
PART 1 21
O1    3    0.156692    0.209642    0.530001    21.00000    0.02315    0.02219 =
          0.01893    0.00227   -0.00526    0.00515
C1    1    0.197695    0.149686    0.544131    21.00000    0.02311    0.01933 =
          0.02257    0.00291   -0.00585    0.00520
C2    1    0.282991    0.125315    0.491233    21.00000    0.03936    0.03109 =
          0.02323    0.00341   -0.00131    0.01347
F1    4    0.360748    0.075978    0.512602    21.00000    0.03664    0.03565 =
          0.03483    0.00716    0.00383    0.01729
F2    4    0.359755    0.171939    0.471788    21.00000    0.04109    0.03438 =
          0.03281   -0.00015    0.01321    0.01417
F3    4    0.210835    0.104067    0.437922    21.00000    0.07243    0.03058 =
          0.03216   -0.01057   -0.01708    0.03014
C3    1    0.086991    0.103824    0.549299    21.00000    0.02692    0.03316 =
          0.05474    0.01002   -0.00086    0.00109
F4    4    0.120410    0.041250    0.544284    21.00000    0.03266    0.03244 =
          0.07659    0.01314    0.00589   -0.00254
F5    4   -0.004747    0.113714    0.499154    21.00000    0.02871    0.04631 =
          0.06699   -0.00018   -0.00756    0.00283
F6    4    0.032053    0.111727    0.606598    21.00000    0.04342    0.04534 =
          0.06816    0.01912    0.01300    0.00142
C4    1    0.278833    0.152099    0.609841    21.00000    0.02997    0.02802 =
          0.02149    0.00544   -0.00704    0.00570
F7    4    0.217441    0.187353    0.652825    21.00000    0.03887    0.04131 =
          0.02281    0.00659   -0.00204    0.00654
F8    4    0.297811    0.093412    0.637836    21.00000    0.05603    0.03390 =
          0.03278    0.01228   -0.01698    0.00691
F9    4    0.395366    0.177026    0.601546    21.00000    0.03231    0.03248 =
          0.03649   -0.00522   -0.01212    0.00157
RESI 0
PART 0

rem the following was inserted by DSR:
SADI 0.02 C1A C2A C1A C3A C1A C4A
SADI 0.02 F1A C2A F2A C2A F3A C2A F4A C3A F5A C3A F6A C3A F7A C4A F8A C4A F9A =
   C4A
SADI 0.04 C2A C3A C3A C4A C2A C4A
SADI 0.04 O1A C2A O1A C3A O1A C4A
SADI 0.04 F1A F2A F2A F3A F3A F1A F4A F5A F5A F6A F6A F4A F7A F8A F8A F9A F9A =
   F7A
SADI 0.1 F1A C1A F2A C1A F3A C1A F4A C1A F5A C1A F6A C1A F7A C1A F8A C1A F9A =
   C1A
```

```
SIMU O1A > F9A
RIGU O1A > F9A
PART 2
O1A   3    0.154632    0.207933    0.527429    11.00000    0.04000
C1A   1    0.191996    0.145825    0.545498    11.00000    0.04000
C2A   1    0.200752    0.140005    0.622166    11.00000    0.04000
F1A   4    0.305148    0.169483    0.649143    11.00000    0.04000
F2A   4    0.201541    0.079089    0.642786    11.00000    0.04000
F3A   4    0.097665    0.169670    0.644788    11.00000    0.04000
C3A   1    0.092807    0.096365    0.514039    11.00000    0.04000
F4A   4    0.064140    0.110338    0.450934    11.00000    0.04000
F5A   4   -0.016859    0.100423    0.544947    11.00000    0.04000
F6A   4    0.135162    0.035786    0.518553    11.00000    0.04000
C4A   1    0.326396    0.135612    0.518646    11.00000    0.04000
F7A   4    0.315964    0.127256    0.453432    11.00000    0.04000
F8A   4    0.383121    0.083316    0.546126    11.00000    0.04000
F9A   4    0.401357    0.186404    0.531978    11.00000    0.04000
AFIX   0
PART 0
rem The end of the DSR entry

rem rem dsr put oc(cf3)3 with o1 c1 c2 c3 on O1_1 C1_1 q15 q14 part 2 -21

RESI 3 CF3
PART 1 31
O1    3    0.081800    0.235411    0.399962    31.00000    0.02269
C1    1    0.033372    0.232537    0.337248    31.00000    0.02263
C2    1    0.146121    0.230044    0.293483    31.00000    0.07563
F1    4    0.113536    0.207456    0.233219    31.00000    0.04423
F2    4    0.245556    0.200608    0.318034    31.00000    0.04443
F3    4    0.177383    0.298455    0.290893    31.00000    0.06590
C3    1   -0.047007    0.289792    0.317142    31.00000    0.06657
F4    4   -0.015523    0.342850    0.348530    31.00000    0.04231
F5    4   -0.064007    0.300531    0.253531    31.00000    0.03919
F6    4   -0.174400    0.269329    0.340816    31.00000    0.06549
C4    1   -0.042812    0.170935    0.325702    31.00000    0.06900
F7    4   -0.111534    0.156135    0.374179    31.00000    0.04207
F8    4   -0.120582    0.172784    0.270319    31.00000    0.05058
F9    4    0.058836    0.122805    0.319484    31.00000    0.06663
RESI 0
PART 0

RESI 2 CF3
O1    3    0.120080    0.336659    0.494426    11.00000    0.01445    0.02176 =
         0.02347   -0.00009   -0.00214   -0.00291
C1    1    0.223789    0.375512    0.504147    11.00000    0.01922    0.02446 =
         0.01675   -0.00034   -0.00606    0.00209
C2    1    0.281319    0.368769    0.575106    11.00000    0.02786    0.03396 =
         0.02473   -0.00516   -0.00826   -0.00253
F1    4    0.352077    0.314955    0.582945    11.00000    0.03615    0.03269 =
         0.04176    0.00669   -0.02389    0.00497
F2    4    0.191896    0.365437    0.617058    11.00000    0.04052    0.05331 =
         0.01801    0.00126    0.00065   -0.01022
F3    4    0.358359    0.417667    0.594043    11.00000    0.03351    0.03715 =
         0.03469   -0.00423   -0.01418   -0.01030
C3    1    0.180032    0.444507    0.492855    11.00000    0.02563    0.02125 =
         0.02837    0.00266    0.00070   -0.00523
F4    4    0.116221    0.466033    0.542783    11.00000    0.03319    0.02567 =
         0.03803   -0.00809    0.00266    0.00136
F5    4    0.276593    0.484828    0.485871    11.00000    0.02781    0.02726 =
```

```
         0.04871    0.00319    0.00259   -0.00680
F6    4    0.100759    0.449528    0.438703    11.00000    0.03898    0.03388 =
         0.03322    0.00908   -0.00968    0.00768
C4    1    0.324256    0.359363    0.456165    11.00000    0.01865    0.02847 =
         0.03315    0.00171    0.00208   -0.00016
F7    4    0.439198    0.385328    0.473759    11.00000    0.01729    0.04404 =
         0.05057   -0.00420    0.00175   -0.00678
F8    4    0.290860    0.379953    0.395614    11.00000    0.03767    0.04288 =
         0.02398    0.00162    0.00874   -0.00142
F9    4    0.342682    0.296329    0.451511    11.00000    0.02300    0.02615 =
         0.05130   -0.01040    0.00470    0.00386
RESI 0
RESI 4 CF3
O1    3   -0.097841    0.261274    0.489484    11.00000    0.01763    0.02418 =
         0.01555   -0.00406    0.00248    0.00104
C1    1   -0.186843    0.282708    0.526803    11.00000    0.01701    0.01847 =
         0.01798    0.00022   -0.00212   -0.00417
C2    1   -0.155278    0.264593    0.600644    11.00000    0.02003    0.03844 =
         0.02341   -0.00570   -0.00223    0.00368
F1    4   -0.031112    0.275566    0.616775    11.00000    0.02552    0.05113 =
         0.02260   -0.00420   -0.00727    0.00468
F2    4   -0.175472    0.202321    0.610260    11.00000    0.03904    0.03977 =
         0.03003    0.01650    0.00263    0.00387
F3    4   -0.222817    0.296838    0.641346    11.00000    0.03175    0.06390 =
         0.01794   -0.00770    0.00409    0.01074
C3    1   -0.200650    0.357301    0.522420    11.00000    0.02037    0.03111 =
         0.02907   -0.00643   -0.00278    0.00165
F4    4   -0.110636    0.386539    0.560147    11.00000    0.02804    0.02847 =
         0.04540   -0.01592   -0.00126   -0.00172
F5    4   -0.189167    0.376459    0.461529    11.00000    0.04719    0.02557 =
         0.03265    0.00720    0.00266    0.00222
F6    4   -0.313803    0.377149    0.540550    11.00000    0.02245    0.03301 =
         0.04168   -0.00621    0.00099    0.00914
C4    1   -0.316216    0.251064    0.503877    11.00000    0.02382    0.03163 =
         0.02487    0.00288   -0.00174    0.00015
F7    4   -0.362398    0.278516    0.447770    11.00000    0.02302    0.04023 =
         0.02897    0.00131   -0.01216    0.00374
F8    4   -0.404458    0.257464    0.546968    11.00000    0.01762    0.05015 =
         0.03179   -0.00160    0.00590   -0.00354
F9    4   -0.301403    0.189114    0.492650    11.00000    0.02542    0.02124 =
         0.04111   -0.00397   -0.00273   -0.00460
RESI 0

HKLF 4

REM  p21c in P2(1)/c
REM R1 =  0.2887 for    7085 Fo > 4sig(Fo)  and  0.3478 for all   10786 data
REM    638 parameters refined using    130 restraints

END

WGHT      0.2000      0.0000

REM Highest difference peak 11.420,  deepest hole -8.938,  1-sigma level  0.653
Q1    1   0.1134  0.0803  0.4526  11.00000  0.05    5.64
Q2    1   0.3271  0.1636  0.4610  11.00000  0.05    5.46
Q3    1  -0.0290  0.1166  0.5154  11.00000  0.05    5.14
Q4    1   0.0765  0.1290  0.6446  11.00000  0.05    5.09
Q5    1   0.1125  0.1302  0.3119  11.00000  0.05    4.77
Q6    1   0.2094  0.2892  0.2890  11.00000  0.05    4.47
```

Q7	1	0.0317	0.1149	0.3283	11.00000	0.05	4.37
Q8	1	0.1222	0.3284	0.2923	11.00000	0.05	4.30
Q9	1	-0.1941	0.2417	0.3472	11.00000	0.05	4.25
Q10	1	-0.1625	0.2875	0.3369	11.00000	0.05	4.22
Q11	1	0.4068	0.1614	0.5611	11.00000	0.05	4.00
Q12	1	0.1228	0.1931	0.2973	11.00000	0.05	3.74
Q13	1	0.0292	0.3051	0.3085	11.00000	0.05	3.37
Q14	1	-0.1075	0.2027	0.3325	11.00000	0.05	3.22
Q15	1	0.3741	0.8104	0.2694	11.00000	0.05	1.70
Q16	1	0.3753	0.5588	0.1961	11.00000	0.05	1.51
Q17	1	0.1501	0.1514	0.2592	11.00000	0.05	1.49
Q18	1	0.1101	0.2008	0.5372	11.00000	0.05	1.47
Q19	1	0.3981	0.5770	0.1743	11.00000	0.05	1.43
Q20	1	0.6544	0.4366	0.3105	11.00000	0.05	1.42

Fragments Included In the Database

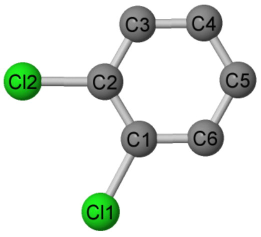

12-dichlorobenz

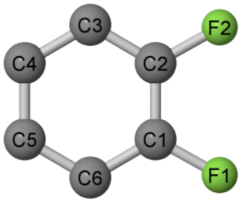

12-difluorobenz

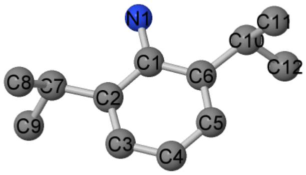

26dpa

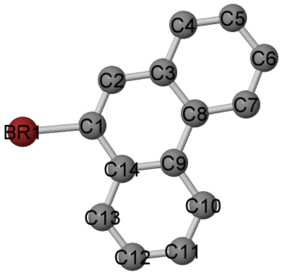

9-bromphen

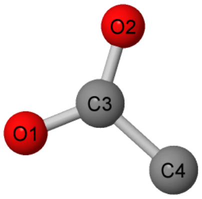

acetate

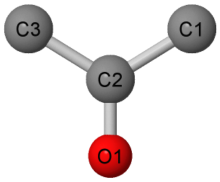

acetone

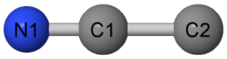

acetonitrile

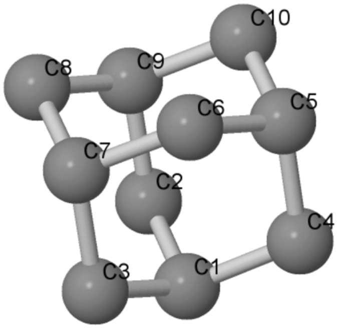

adamantane

Jmol

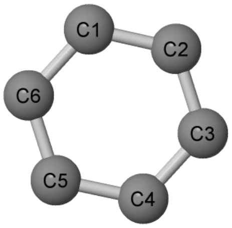

benzene

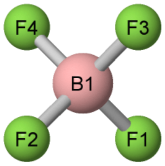

bf4

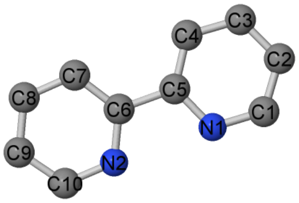

bipyridine

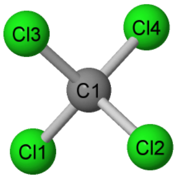

ccl4

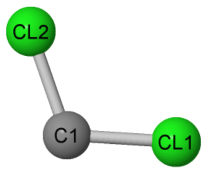

ch2cl2

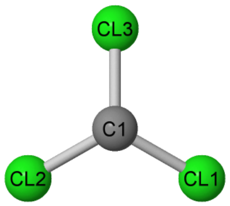

chcl3

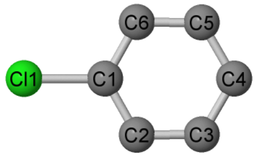

chlorobenz

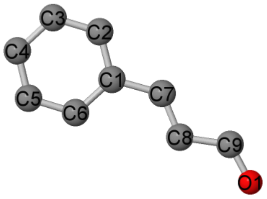

cinnamald

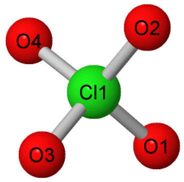

clo4

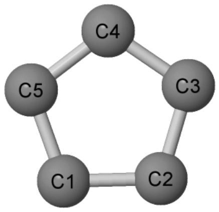

cp

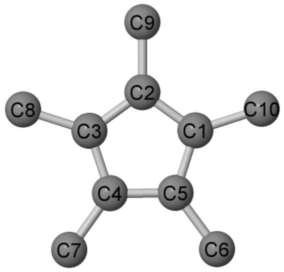

cpstar

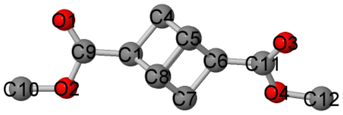

cubane_dicarbox

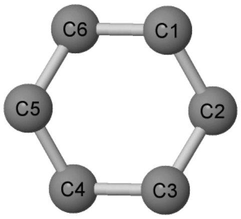

cyclohexane

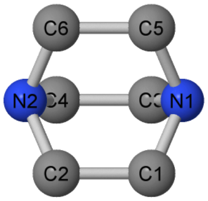

dabco

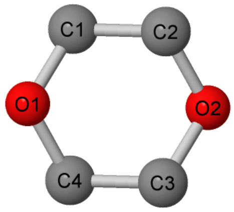

diox

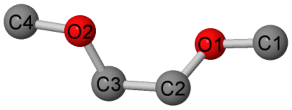

dme-free

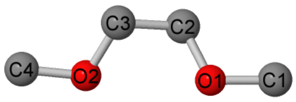

dme

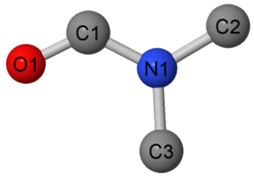

dmf

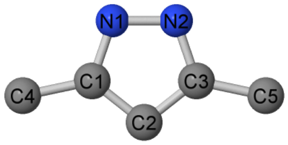

dmpz

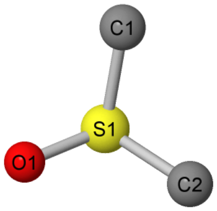

dmso

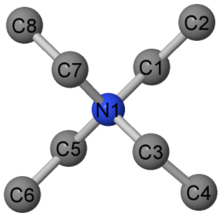

et4n+

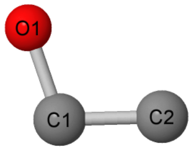

ethanol

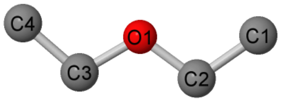

ether

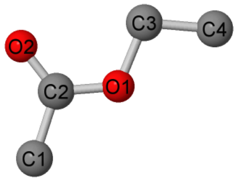

ethylac

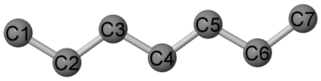

heptane

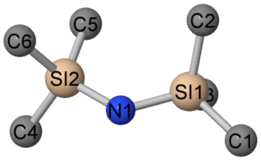

hexa

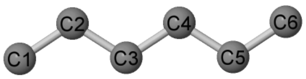

hexane

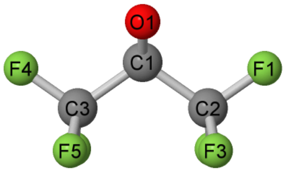

hfipo

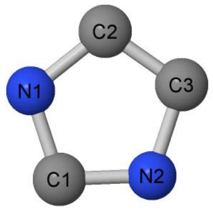

imidazole

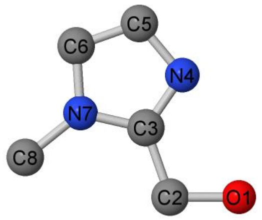

mimme

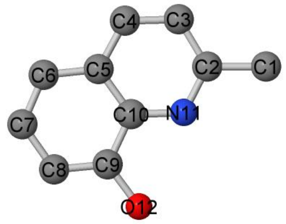

mquinolinol

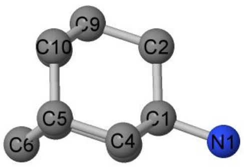

n-adamantane

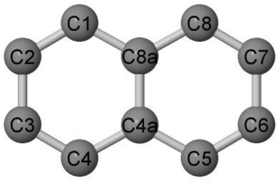

napht

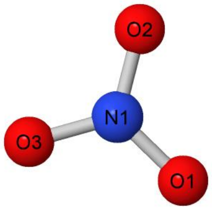

nitrate

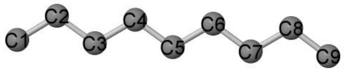

nonane

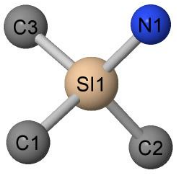

nsime3

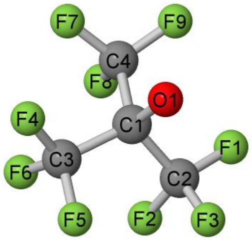

oc(cf3)3

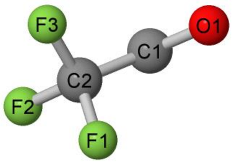

och2cf3

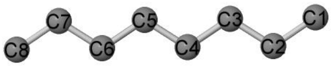

octane

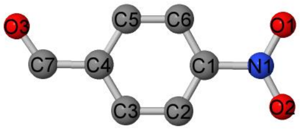

p-toluidin

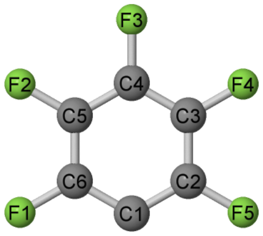

pentafl

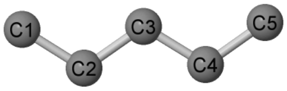

pentane

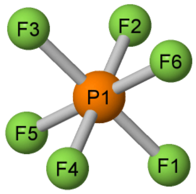

pf6

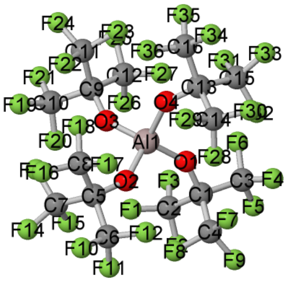

pfanion

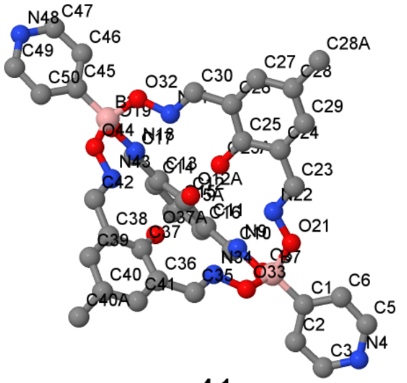

pm41

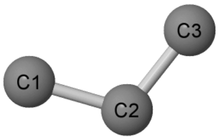

propane

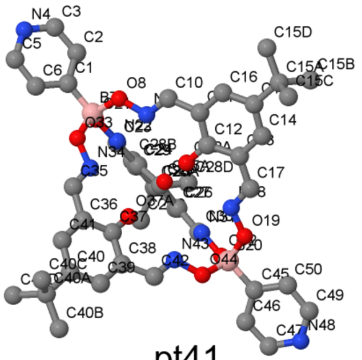

pt41

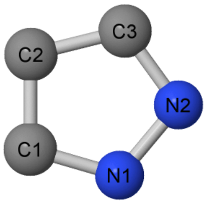

pyrazole

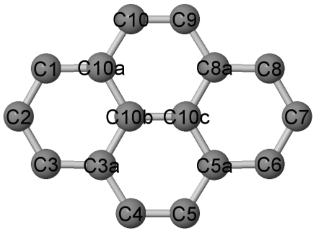

pyrene

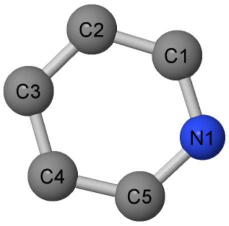

pyridine

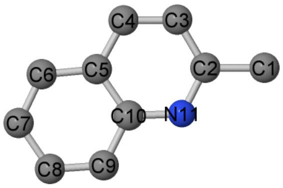

quinaldine

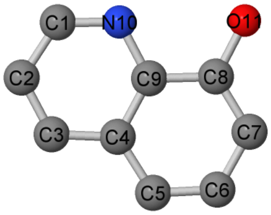

quinolinol

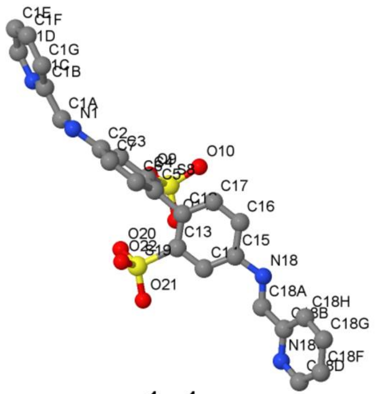

s1p1

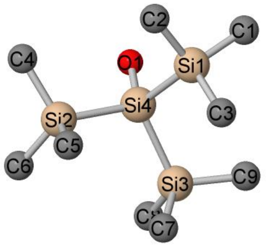

supersilyle

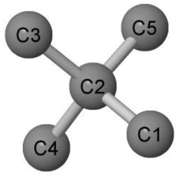

tbu-c

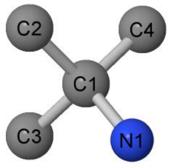

tbu-n

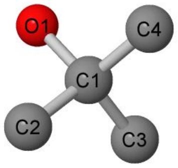

tbu-o

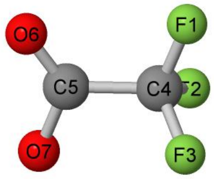

tfa

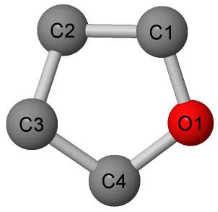

thf

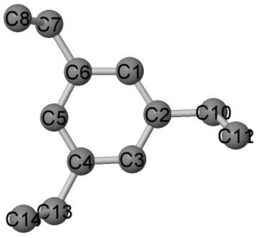

tip

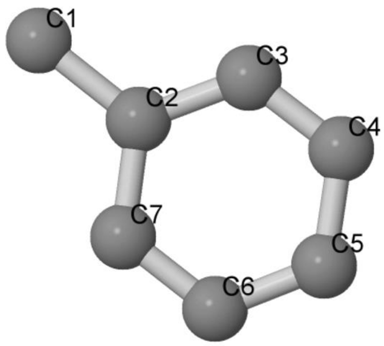

toluene

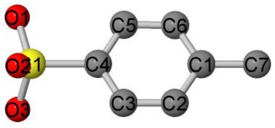

tosylate

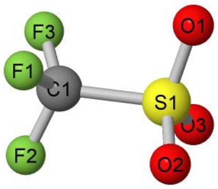

triflate

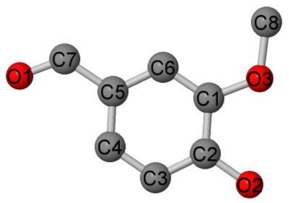

vanillin

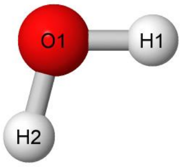

water

Pictures were automatically drawn using Jmol (*Jmol: an open-source Java viewer for chemical structures in 3D.*) with the following options:

```
font label 55 sanserif
set label ON
label %a
color labels black
color bonds lightgrey
translate 25 25 25
zoom 0
set axes OFF
set showUnitcell FALSE
set bondRadiusMilliAngstroms 100
background white
rotate BEST
set ambient 40
quit
```

References

Bihlmeier, A., Gonsior, M., Raabe, I., Trapp, N. & Krossing, I. (2004). *Chem. Eur. J.* **10**, 5041-5051.

Dolomanov, O. V., Bourhis, L. J., Gildea, R. J., Howard, J. A. K. & Puschmann, H. (2009). *J. Appl. Cryst.* **42**, 339-341.

Guzei, I. A. (2014). *J. Appl. Cryst.* **47**, 806-809.

Hagberg, A., Schult, D. & Swart, P. (2013). *NetworkX.*

ImageMagick, (2014). http://www.imagemagick.org.

Jmol: an open-source Java viewer for chemical structures in 3D., http://www.jmol.org/

Köchner, T., Engesser, T. A., Scherer, H., Plattner, D. A., Steffani, A. & Krossing, I. (2012). *Angew. Chem.* **124**, 6635-6637.

Krossing, I. (2001). *Chem. Eur. J.* **7**, 490-502.

Kymoto, S. (2014). *Inno Script Studio,* https://www.kymoto.org/products/inno-script-studio.

Lichtenthaler, M. R., Higelin, A., Kraft, A., Hughes, S., Steffani, A., Plattner, D. A., Slattery, J. M. & Krossing, I. (2013). *Organometallics* **32**, 6725-6735.

Pascu, M., Marmier, M., Schouwey, C., Scopelliti, R., Holstein, J. J., Bricogne, G. & Severin, K. (2014). *Chem. Eur. J.* **20**, 5592-5600.

Smart, O. & Womack, T. (2014). *Grade Web Server, Global Phasing Ltd.,* http://grade.globalphasing.org/.

Spek, A. (2009). *Acta Cryst.* **D65**, 148-155.
